# Experimental evidence for the beneficial effects of low-concentration ozone

**DOI:** 10.4103/mgr.MEDGASRES-D-25-00198

**Published:** 2026-09-12

**Authors:** Flavia Carton, Carlo Pellicciari, Gabriele Tabaracci, Manuela Malatesta

**Affiliations:** 1 Department of Neurosciences, Biomedicine and Movement Sciences, University of Verona, Verona, Italy; 2 Center for Medical Sciences (CISMed), University of Trento, Trento, Italy; 3 Department of Cellular, Computational and Integrative Biology (CIBIO), University of Trento, Trento, Italy; 4 Department of Biology and Biotechnology, University of Pavia, Pavia, Italy; 5 San Rocco Clinic, Montichiari (BS), Italy

**Keywords:** anti-inflammatory, antioxidant, antitumor, apoptosis, autophagy, cell differentiation, cell proliferation, dose-dependent effect, eustress, oxidative stress, regeneration

## Abstract

Ozone (O_3_), a molecule consisting of three oxygen atoms, is an unstable compound whose clinical application as a complementary therapy continues to expand. Although numerous clinical studies support its therapeutic potential, the molecular basis of its effects has remained poorly understood. This review summarizes the current knowledge on the cellular and molecular mechanisms activated by low-concentration O_3_. Central to its action is the induction of mild oxidative stress by low concentrations of O_3_, which triggers an antioxidant response mediated through nuclear factor erythroid 2-related factor 2, enhancing cell survival. Additionally, low-concentration O_3_ exhibits antiinflammatory properties by modulating cytokine expression, inhibiting metalloproteinases, and activating macrophages. The regenerative potential of low-concentration O_3_ may be attributed to the stimulation of fibroblasts, chondrocytes, and osteoblasts. Moreover, the antitumor effects of low-concentration O_3_ in cancer cell lines and animal models appear to be linked to the inhibition of cell proliferation and migration, as well as proapoptotic activity. More research is needed to determine the best treatment methods, long-term effectiveness, and standardized clinical procedures for ozone therapy in specific diseases. This review provides a detailed reference for understanding how O_3_ therapy works.

## Introduction

Ozone (O_3_) is a highly unstable chemical compound made of three oxygen atoms that has a half-life of approximately 40 minutes at 20°C and rapidly decomposes into oxygen. With a mean concentration of 1 mg/m^3^ in the atmosphere, O_3_ is unevenly distributed, with its maximum concentration occurring 20 to 25 km from the Earth’s surface, where a thin O_3_ layer exerts a vital protective role for all living organisms, as it absorbs most of the ultraviolet sun radiation.^1^

O_3_ was first detected in 1785 by the Dutch physician Martinus van Marum, who noticed a typical smell after an electric spark passed through the air.^2^ In 1839, O_3_ was identified as a distinct chemical substance by Christian Friedrich Schönbein,^3^ who named it ozone from the Greek word őζειν (ozein that means to smell). Schönbein^4^ was the first to demonstrate the oxidizing power of O_3_, which can react with inorganic and organic compounds and is thus potentially harmful to animal and human health, especially for breath.^5^ The disinfectant action of O_3_ against microorganisms was discovered by Fox^6^ and Kellogg,^7^ and its use in medical practice was made possible when the Tesla Ozone Company in 1900 produced and sold O_3_ generators based on Nikola Tesla’s US Patent No. 568,177 (September 22, 1896).

In the following years, it was practically established that O_3_ may have significant applications in treating various pathological conditions (e.g., parodontitis, infected wounds, chronic colitis, and proctitis), but the use of O_3_ as an adjunctive medical treatment became increasingly accepted and extensively applied only after O_3_-resistant plastics were produced (in the 1950s), and O_3_ generators that accurately measure gas concentrations were available on the market (in the 1990s).^8^,^9^,^10^,^11^,^12^,^13^,^14^

Medical O_3_ is typically administered as a gaseous O_2_–O_3_ mixture or as ozonated oil or water; administration may be either systemic (by blood-stream or rectal insufflation or orally) or local (by injection or topical application). According to Viebahn-Hänsler et al.,^15^,^16^ O_3_ treatments are presently based on the low-dose concept, as it was demonstrated that positive effects may occur only within a relatively narrow range of low concentrations (from 10 to 50 µg O_3_/mL O_2_) when moderate oxidative stress is induced.^17^ In fact, strong oxidative stress may have deleterious effects on cells, tissues and organs (the so-called distress), whereas mild oxidative stress may activate defensive molecular pathways; under this condition, defined as eustress,^18^,^19^ a protective response is induced through changes in gene expression that stimulate antioxidant and anti-inflammatory reactions without causing cell damage.^20^,^21^ Currently, low-dose O_3_ is successfully administered, often as an adjuvant treatment, in many medical fields, such as angiology,^22^ dentistry,^23^,^24^ dermatology,^25^,^26^ neurology,^27^ orthopedics and rheumatology.^28^,^29^

Although the beneficial effects of O_3_ have been shown in several clinical observational studies,^22^,^23^,^24^,^25^,^26^,^27^,^28^,^29^ the underlying molecular mechanisms have long been unclear. The goal of the present review is to offer an overview of the present knowledge on the mechanisms underlying the cellular and molecular processes that occur following low O_3_ concentration. We aimed to provide a comprehensive understanding of the diverse effects of ozone therapy on organisms, and to examine the related cell and molecular processes.

## Search Strategy

The scientific literature on medical O_3_ was reviewed, focusing on articles published since the 1990s, when the first articles on the subject were published. The search was carried out in the PubMed, Web of Science, Embase, and Cochrane Library databases; only articles in English based on low O_3_ concentrations were considered, while investigations using high O_3_ concentrations were excluded. Articles dealing with experimental studies performed on systems *in vitro*, *ex vivo* (on animals and humans) and *in vivo* (on animal models) were reviewed, whereas clinical studies on humans and animals were excluded. Special attention was given to articles investigating the cellular and molecular mechanisms driven by O_3_ administration; purely descriptive reports were only marginally considered.

## Antioxidant Mechanisms of Low-Concentration Ozone

Low concentrations of O_3_ activate the body’s endogenous antioxidant system, initiating a beneficial adaptive response at multiple levels.

### Antioxidant mechanisms

The antioxidant effects of O_3_ are largely based on the activation of nuclear factor erythroid 2-related factor 2 (Nrf2). In an *in vitro* study on endothelial cells, the addition of human serum treated with low-concentration O_3_ increased the nuclear level of Nrf2^30^ and the cellular level of heme oxygenase-1 (HO-1). Galiè et al.^31^ provided a mechanistic explanation for this effect, as a subsequent *in vitro* investigation demonstrated that low-concentration O_3_ promotes the translocation of Nrf2 to nuclear active transcription sites, thus inducing the expression of antioxidant response element-driven genes, which encode many cytoprotective and detoxifying molecules, including HO-1. This study also demonstrated that the expression of the specific Nrf2 inhibitor Kelch-like ECH-associated protein 1 restrains the O_3_-induced activation of antioxidant response elements. The role of the Kelch-like ECH-associated protein 1-Nrf2 pathway in triggering the cell antioxidant response following exposure to low concentrations of O_3_ was thus clearly established.

Many experimental studies support these findings. Systemic O_3_ treatment improved pancreatic function in a rat model of pancreatic damage by activating antioxidant and antiapoptotic processes through an increase in Nrf2 and glutathione-S-transferase activity and a reduction in 4-hydroxynonenal and poly(adenosine diphosphate ribose) polymerase-1 expression.^32^ O_3_ has been proved to protect the myocardium by activating Nrf2 both *in vivo* (mouse ischemia–reperfusion) and *in vitro* (cardiomyocyte hypoxia–reoxygenation) and to ameliorate neuronal damage in rats. Nrf2 activation inhibits ferroptosis (a type of regulated oxidative cell death) and increases the expression of antioxidant factors such as solute carrier family 7 member 11 and glutathione peroxidase (GPx)4.^33^,^34^ A particular type of programmed cell death is parthanatos; it occurs after cerebral ischemia–reperfusion injury, and oxidative stress is a main contributing factor.^35^ In an *in vitro* model of cell reperfusion injury as well as in a mouse model of ischemic stroke *in vivo*, O_3_ reduced neuronal parthanatos through the activation of Nrf2 and peroxisome proliferator-activated receptor γ, thus inhibiting reactive oxygen species (ROS) production.^36^

O_3_ has been shown to mitigate skeletal muscle ischemia–reperfusion injury in a rat model by modulating the Nrf2/HO-1 pathway.^37^ O_3_ was also able to alleviate oxidative stress injury in renal transplantation by activating Nrf2/HO-1 signaling pathways and inhibiting apoptosis in tubular epithelial cells.^38^ O_3_ reduced kidney injury in rats by modulating Nrf2 and nuclear factor kappa-light-chain-enhancer of activated B cells (NF-κB), thus increasing the expression of superoxide dismutase (SOD) and catalase (CAT) and reducing the levels of glutathione (GSH) and malondialdehyde (MDA), protein carbonyl (PCO), and some inflammatory cytokines (interleukin (IL)-1β, IL-6, tumor necrosis factor (TNF)-α, and intercellular adhesion molecule-1).^39^ Furthermore, in a rat model of lung ischemia–reperfusion injury, Wang et al.^40^ reported that O_3_ exerts a protective effect by increasing Nrf2 expression while simultaneously decreasing the levels of nucleotide-binding oligomerization domain-like receptor family pyrin domain containing 3 (NLRP3), apoptosis-associated speck-like protein containing a caspase recruitment domain (ASC), procaspase-1, caspase-1 and IL-1β.

Studies on various oxidative stress models have shown that O_3_ treatment mitigates oxidative damage through molecular pathways other than Nrf2. In rats, O_3_ significantly reduced the severity of experimentally induced seizures^41^ and protected the liver from oxidative damage^42^ by activating A1 adenosine receptors, thus promoting cellular signaling to preserve the cellular redox balance. Studies on kidney^43^,^44^ and skeletal muscle^45^ undergoing ischemia–reperfusion injury have shown that O_3_ exerts its antioxidant effects through the production of nitric oxide (which is known to modulate HO-1 activity) and endothelial nitric oxide synthase (NOS) and the increase in inducible NOS (iNOS) expression. An *in vivo* and *in vitro* study of rat kidney ischemia–reperfusion also demonstrated that O_3_ protects renal tissue from apoptosis and oxidative stress by inhibiting the activation of mitogen-activated protein kinase.^46^

Some studies have shown the ability of O_3_ to modulate mitochondrial activity, which is crucial for the generation of ROS and the maintenance of the oxidant/antioxidant balance in cells. O_3_ treatment partially restored impaired mitochondrial function in rats after endotoxemic shock, stimulating the expression of respiratory enzymes, succinate dehydrogenase and adenosine triphosphatase, while diminishing the activity of lactate dehydrogenase.^47^ In a rat model of hearing loss, O_3_ treatment protected brain and cochlear mitochondria by enhancing the activity of antioxidant enzymes, such as CAT, SOD, and GPx, and decreasing the level of MDA.^48^ Moreover, mitochondria isolated from animals treated with O_3_ showed decreased ROS production and cytochrome C release, whereas the membrane potential and adenosine triphosphate (ATP) levels increased, and noise-induced mitochondrial swelling was reversed.^48^ ROS production, mitochondrial swelling, and the association of Nrf2 with the outer mitochondrial membrane were also evaluated in murine myoblasts treated with O_3_ at different concentrations, revealing that a low dose of O_3_ did not alter mitochondrial structure and reduce ROS production.^49^ In the mouse liver, O_3_ reduced mitochondrial respiration (complexes I and II/III), reduced GSH and increased GPx and SOD activity, thus preventing mitochondrial ROS production without affecting energy charge.^50^

Methodological details of the experimental investigations reviewed in this chapter are reported in **Additional Table 1**. **Additional Table 2** summarizes the molecules involved in the antioxidant properties of low-concentration O_3_, and **Figure 1** shows a schematic representation of the recognized molecular pathways responsible for the antioxidant effects of O_3_.

**Additional Table 1:** The experimental investigations on the cellular and molecular mechanisms for antioxidant effect of low O_3_ concentration

Experimental model	Target organ/tissue	O_3_ treatment	Administration route	Reference
***In vitro***				
Human EA.hy 926 endothelial cells		20–80 µg O_3_/mL O_2_; Single treatment	Medium	1
Human HeLa adenocarcinoma cells		10–35 µg O_3_/mL O_2_; Single treatment	Medium	2
Mouse C2C12 myoblasts		10, 20 µg O_3_/mL O_2_; Single treatment	Medium	3
Mouse C57 cardiomyocytes		O_3_ flow rate of 2 L/min for 4 h	Medium	4
***Ex vivo***				
Human healthy donors	Blood	2.2–108 µg O_3_/mL O_2_; Single treatment	In syringe, continuous gas flow	5
Human healthy donors	Blood	35–100 µg O_3_/mL O_2_; Single treatment	In syringe	6
Human healthy donors	Blood	20–80 µg O_3_/mL O_2_; Single treatment	In syringe	7
Human healthy donors	Blood	5–800 µg O_3_/mL O_2_; Single treatment	In syringe	8
Human healthy donors	Blood	15, 30 μg O_3_/mL O_2_; Single treatment	In bag	9
Mouse	Adipose tissue	10–100 µg O_3_/mL O_2_; Single treatment	Medium	10
Sheep	Blood	5, 10 μg O_3_/mL O_2_; Single treatment	Continuous gas flow	11
***In vivo***				
Rat	Liver	3 g/kg/day; Daily for 4 wk	Oral	12
Rat	Blood	150 µg O_3_/mL O_2_; 3 g/kg/d Daily for 3 wk	Intraperitoneal	13
Rat model of intestinal ischemia-reperfusion injury	Small intestine	1 mg/kg; Single infusion	Intraperitoneal	14
Rat model of ischemia-reperfusion injury	Testicle	4% O_3_; 1 mg/kg Daily for 7 d	Intraperitoneal	15
Rat model of lesion caused by *Leishmania amazonensis*	Skin	0.1 mL; Daily for 30 d	Topical	16
Rat model of septic shock	Liver, lung	10–50 O_3_/mL O_2_; Daily for 5 d	Intraperitoneal	17
Rat model of sciatic nerve injury	Sciatic nerve	35–40 µg O_3_/mL O_2_; 5 mL; Daily for 2 mon	Intraperitoneal	18
Rat model of aging	Liver, kidney	5% O_3_; 0.6 mg/kg; Twice weekly for 3 mon	Rectal insufflation	19
Rat model of aging	Brain	1–120 µg O_3_/mL O_2_; 0.6 mg/kg; Twice weekly for 3 mon	Rectal insufflation	20
Rat model of aging	Heart, hippocampus	0.6 mg O_3_/kg; 5 mL; Twice weekly for 3 mon, then once a week till the age of 15 mon	Rectal insufflation	21
Rat model of aging	Cerebellum	80 mg O_3_/mL O_2_; 0.7 g/kg; 3 times weekly for 8 wk	Intraperitoneal	22
Rat model of streptozotocin-induced pancreatic damage	Pancreas	150 µg O_3_/kg; Daily for 7 d	Intraperitoneal	23
Rat model of cerebral ischemia-reperfusion injury	Brain	40 µg O_3_/mL O_2_; 2 mg/kg; Daily for 5 d	Intraperitoneal	24
Rat model of kidney transplantation	Kidney	50 µg O_3_/mL O_2_; 1 mg/kg; Daily for 15 treatments	Rectal insufflation	25
Rat model of tubulointerstitial injury	Kidney	50 µg O_3_/mL O_2_; 1.1 mg/kg	Rectal insufflation	26
Rat model of lung ischemia-reperfusion injury	Lung	100 µg O_3_/kg; 2 mL; Daily for 10 d	Intraperitoneal	27
Rat model of liver ischemia-reperfusion injury	Liver	50 µg O_3_/mL O_2_; 1 mg/kg; Daily for 15 d	Rectal insufflation	28
Rat model of renal ischemia-reperfusion injury	Kidney	50 µg O_3_/mL O_2_; 1 mg/kg; Daily for 15 d	Rectal insufflation	29
Rat model of renal ischemia-reperfusion injury	Kidney	50 µg O_3_/mL O_2_; 1 mg/kg; Daily for 15 d	Rectal insufflation	30
Rat model of skeletal muscle ischemia-reperfusion injury	Tibialis anterior muscle	60 µg O_3_/mL O_2_; 0.7 mg/kg; 6 treatments	Intraperitoneal	31
Rat model of renal ischemia/reperfusion injury	Kidney	50 µg O_3_/mL O_2_; 1–2 mg/kg; Daily for 15 d	Rectal insufflation	32
Rat model of endotoxemic shock	Liver, kidney, lung, heart.	54 mg O_3_/dm^3^; 150 µg/kg; Daily for 10 d	Intraperitoneal	33
Rat model of noise-induced hearing loss	Brain, cochlear	30 µg O_3_/mL O_2_; 1 mL; Daily for 14 d	Intravenous	34
Rat model of partial hepatectomy after ischemia-reperfusion injury	Liver	60 µg O_3_/mL O_2_; 1.2 mg/kg; Daily for 5 d	Intraperitoneal	35
Mouse model of induced seizures	Brain	20 µg O_3_/mL O_2_; 1–1.5 mL; 1 mg/kg; Daily up to 20 d	Rectal insufflation	36
Mouse	Liver	50 µg O_3_/mL O_2_; 80 mL/kg; Daily for 5 d	Intraperitoneal	37
***In vivo and in vitro***				
Rat model of spinal cord injury; Mouse RAW264.7 macrophage Mouse primary spinal cord neurons	Spinal cord	Once for 3 d	Reinfusion of ozonated blood	38
Rat model of skeletal muscle ischemia-reperfusion injury; Rat L6 myoblasts	Skeletal muscle	15–60 μg O_3_/mL O_2_; 2 mL ozonated blood Daily for 3 d (*in vivo*) or single treatment (*in vitro*)	Intravenous, medium	39
Mouse model of ischemic stroke; Human SH-SY5Y neuroblastoma cells.	Brain	20 mg O_3_/mL O_2_; 1.5 mg/mL; Single treatment	Intravenous, medium	40
Mouse model of ischemia-reperfusion injury; Rat H9c2 cardiomyoblasts	Heart	25 µg O_3_/mL O_2_; 2 mL Daily for 5 d	Intraperitoneal, medium	41
Mouse model of Leishmaniasis lesions; Promastigotes of *L. amazonensis*	Skin	1–30 µg O_3_/mL O_2_; 50 mL; Daily for3 d per week for 30 d	Intraperitoneal, topical, medium	42

*L. amazonensis*: *Leishmania amazonensis*; O_3_: ozone.

**Additional Table 2:** Molecule modulation of low O_3_ concentration and accounting for antioxidant effects

Experimental model	Molecules	Reference
***In vitro***		
Human endothelial and epithelial cells	Nrf2, Keap1, HO-1	1 2
***Ex vivo***		
Human blood	H_2_O_2_, GSH, GSSG, formate, allantoine, acetoacetate and acetate, pyruvate, lactic, pyruvic acid, LOPs, lysophospholipids, oxidized phosphatidylcholines, dihydroxyvitamin D3, biliverdin, bilirubin, pyroglutamic acid	3 4 5 6 7
Mouse adipose tissue	Nrf2, HO-1	8
Sheep blood	Carbohydrate, lipid, amino acid and nucleotide metabolites	9
***In vivo***		
Rat, mouse, rabbit, guinea pig	SOD, CAT, GPx, GSH, MDA, PCO	10 11 12 13 14 15 16 17 18 19 20 21 22 23 24 25 26 27 28 29 30 31 32 33 34 35 36 37 38 39 40 41 42 43 44 45 46 47 48 49 50 51 52 53
Rat	LDL-C, apolipoprotein A-I, paraoxonase	54
	SOD, GPx, GSH, MDA, PCO, ATP/ADP ratio, mitochondrial complex I and IV, NOS, iNOS, Na^+^, K^+^-ATPase, caspase-3	55 56 57 58
	Nrf2, GST, 4-HNE, PARP-1	59
	Nrf2, HO-1	60 61
	Nrf2, NF-κB, SOD, CAT, GSH, MDA, PCO, IL-1β, IL-6, TNF-α, ICAM-1	62
	Nrf2, NLRP3, ASC, procaspase-1, caspase-1, IL-1β	63
	A1 adenosine receptors	64 65
	NO, NOS, iNOS	66 67
	Mitochondrial succinate dehydrogenase, ATPase, LDH	68
	CAT, SOD, GPx, MDA, mitochondrial ROS, cytochrome c and ATP	69
Mouse	Nrf2, PPARγ	70
	Mitochondrial complexes I and II/III, GSH, GPx, SOD	71
***In vivo* and *in vitro***		
Rat, mouse, rat cardiomyocytes	Nrf2, Slc7a11, GPx4, miR200c, FOXO3	72 73 74
Rat, rat kidney epithelial cells	MAPK	75
Mouse, mouse macrophages	AST, GST, LDH, NO	76 77

4-HNE: 4-Hydroxynonenal; ADP: adenosine diphosphate; AMPK: adenosine monophosphate-activated protein kinase; ARE: antioxidant response element-driven genes; ASC: apoptosis-associated speck-like protein containing a caspase recruitment domain; AST: aspartate aminotransferase; ATP: adenosine triphosphate; C1q: complement component 1q; CAT: catalase; COX-2: cyclooxygenase-2; FOXO3: Fork-head box O3; GPx: glutathione peroxidase; GSH: reduced glutathione; GSSG: glutathione disulfide; GST: glutathione-S-transferase; H_2_O_2_: hydrogen peroxide; HMGB1: high-mobility group box-1; HO-1: heme oxygenase-1; HSP70: heat shock protein 70; ICAM-1: intercellular adhesion molecule-1; IL: interleukin; iNOS: inducible nitric oxide synthase; JAK2: Janus kinase 2; Keap1: Kelch-like ECH associated protein 1; LDH: lactate dehydrogenase; LDL-C: low-density lipoproteins cholesterol; LOP: lipid oxidation product; MAPK: mitogen-activated protein kinase; MCP-1: monocyte chemoattractant-1; MDA: malondialdehyde; MerTK: MER proto-oncogene tyrosine kinase; miR200c: microRNA encoded by the MIR200C gene; MMP: matrix metalloproteinase; NF-κB: nuclear factor kappa-light-chain-enhancer of activated B cells; NLRP3: nucleotide-binding oligomerization domain-like receptor family pyrin domain containing 3; NO: nitric oxide; NOS: nitric oxide synthase; Nrf2: nuclear factor erythroid 2-related factor 2; O3: ozone; PARP-1: poly(adenosine diphosphate-ribose) polymerase-1; PCO: protein carbonyl; PPARγ: peroxisome proliferator-activated receptor γ; ROS: reactive oxygen species; Slc7a11: solute carrier family 7 member 11; SOD: superoxide dismutase; TNF-α: tumor necrosis factor α.

**Figure 1:**
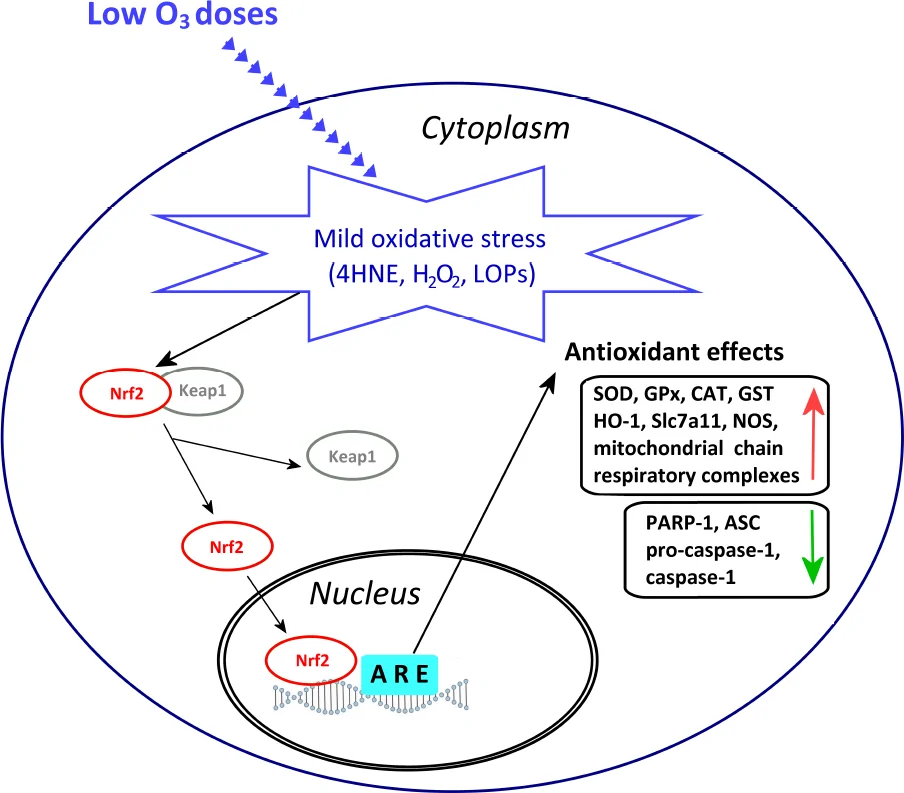
The molecular pathways involved in the antioxidant effects of low-concentration O_3_. Created with Jasc Paint Shop Pro (version 7.04). 4-HNE: 4-Hydroxynonenal; ARE: antioxidant response element-driven gene; ASC: apoptosis-associated speck-like protein containing a caspase recruitment domain; CAT: catalase; GPx: glutathione peroxidase; GST: glutathione-S-transferase; H_2_O_2_: hydrogen peroxide; HO-1: heme oxygenase-1; Keap1: Kelch-like ECH associated protein 1; LOP: lipid oxidation product; NOS: nitric oxide synthase; Nrf2: nuclear factor erythroid 2-related factor 2; PARP-1: poly(adenosine diphosphate ribose) polymerase-1; Slc7a11: solute carrier family 7 member 11; SOD: superoxide dismutase.

### Antioxidant response in blood

It has been demonstrated that human blood treated *ex vivo* with low concentrations of gaseous O_3_ transiently undergoes an increase in ROS, such as hydrogen peroxide and 4-hydroxynonenal, while a decrease in GSH and an increase in its oxidized form occur, indicating that safe oxidative stress is ultimately induced, resulting in an antioxidant response.^8^,^51^,^52^ These findings were confirmed and extended by metabolomic studies on blood ozonated *ex vivo* that revealed molecular modifications related to the antioxidant and anti-inflammatory response as well as to energy production. In fact, the results revealed an increase in formate, allantoine, acetoacetate and acetate, and a decrease in pyruvate,^53^ an increase in lactic and pyruvic acid, lipid oxidation products, lysophospholipids, oxidized phosphatidylcholines, dihydroxyvitamin D3 and biliverdin,^54^ a decrease in bilirubin and pyroglutamic acid,^54^ and modifications in the content of carbohydrate, lipid, amino acid and nucleotide metabolites.^55^

When ozonated oil was orally administered to the rats, the levels of low-density lipoproteins and cholesterol decreased, whereas the ferric ion reduction ability (an index of the antioxidant capacity of blood) and sulfhydryl content (an important antioxidant group) increased. Moreover, apolipoprotein A-I (the primary high-density lipoprotein) was stabilized, while the activity of paraoxonase-1 (an important antioxidant enzyme occurring in high-density lipoproteins) increased, thus positively influencing the functionality of high-density lipoproteins.^56^

### Antioxidant response in animal models of pathological conditions

*In vivo* studies on animal models subjected to various stressors have demonstrated that O_3_ upregulates endogenous antioxidant systems (i.e., SOD, CAT and GPx), increases the levels of non-enzymatic antioxidants (particularly GSH), and improves the cellular redox balance, thus exerting an organ-protective effect on the liver,^57^,^58^,^59^,^60^,^61^,^62^ kidney,^63^,^64^,^65^,^66^ heart,^67^ urinary bladder,^68^ testis,^69^,^70^,^71^ intestine,^59^,^72^,^73^ stomach,^74^ esophagus,^75^ pancreas,^76^,^77^ lung,^58^,^78^ nervous tissue,^79^ and skin.^80^

Interestingly, the aberrant expression of miR-200c (a microRNA encoded by the *MIR200C* gene) by endothelial cells is induced by ROS in ischemic heart disease,^81^ and O_3_ was found to alleviate ischemia-reperfusion damage in mouse cardiomyocytes by regulating the miR-200c/Fork-head box-O3 axis.^82^

O_3_ antioxidant properties play a key role in regeneration across various tissues and organs. Post-surgical treatment with O_3_ increased the levels of GPx, SOD and CAT while decreasing the level of the end product of membrane lipid peroxidation, MDA, thus improving the functional and structural recovery of the sciatic nerve in rats.^83^ In a rat model of spinal cord injury, O_3_ promoted functional recovery by decreasing MDA levels and increasing SOD levels and by lowering the levels of pro-inflammatory cytokines.^84^ A study performed on zebrafish revealed that ozonated oil improved the healing of skin and fin wounds by decreasing ROS levels.^85^ Moreover, the ability of the drug Glucantime® to treat leishmaniasis lesions was ameliorated by the association of O_3_ with accelerated wound healing, increased levels of glutathione-S-transferase, and reduced levels of nitric oxide, aspartate aminotransferase and lactate dehydrogenase.^86^,^87^

O_3_-driven antioxidant processes also play a primary role in adipose tissue preservation *ex vivo*. O_3_ treatment increased Nrf2 protein and *HO-1* gene expression in explanted murine adipose tissue maintained in culture; concurrently, the culture medium reduced glycerol efflux and increased glutamate uptake, suggesting metabolic shifts that support cellular function.^88^

### Antioxidant response in aging

Redox state imbalance is associated not only with experimental or pathological conditions but also with physiological aging. O_3_ has been shown to limit age-related oxidative stress in the kidneys and liver of rats by reducing the levels of MDA and mitochondrial PCO, restoring normal GSH and GPx levels and decreasing lipofuscin deposition. The same effect, albeit less pronounced, was achieved by treating aged rats with O_3_ for 1 month.^89^ Moreover, rats treated with O_3_ for 12 months experienced an improvement in age-related cerebral cortex alterations because of the normalization of the GSH content, the ATP-to-adenosine diphosphate ratio, and the activities of mitochondrial SOD and complex IV (cytochrome-c oxidase). The same study also revealed that O_3_ administration increases the glutathione redox index and the activity of complex I and NOS in mitochondria and increases mitochondrial PCO and MDA levels.^90^ In the heart and hippocampus of aged rats, O_3_ decreased the levels of MDA, PCO, and GSH; normalized GPx activity; reversed the age-related decreases in ATP levels and the ATP-to-adenosine diphosphate ratio; restored Na^+^, K^+^-adenosine triphosphatase activity in the hippocampus; and increased cytosolic Ca^2+^ levels in the heart, thus reducing oxidative stress and improving energy metabolism.^91^ O_3_ treatment also reduced oxidative stress and apoptosis in aged rats by downregulating iNOS and the pro-apoptotic factor caspase-3. Moreover, O_3_-treated rats exhibited increased neurogenesis, as indicated by increased levels of the neurogenetic marker, microtubule-associated protein 2, and an increased number of Purkinje cells, in parallel with a decrease in the expression of the astrocyte marker, glial fibrillary acidic protein.^92^

## Anti-Inflammatory Mechanisms of Low-Concentration Ozone

Low-concentration O_3_ exerts anti-inflammatory effects through several mechanisms that induce a shift in the cytokine balance from pro-inflammation to anti-inflammatory effects.

### Anti-inflammatory mechanisms

There is evidence that O_3_ treatment can attenuate inflammatory responses by modulating/downregulating different pathways, such as Toll-like receptor 4 (TLR4)-NF-κB, which is responsible for the control of many pro-inflammatory genes. O_3_ reduced inflammatory injury in a rat model of chronic kidney disease by suppressing the expression of TLR4 and phosphorylated NF-κB/p65 and significantly reducing the expression of pro-inflammatory cytokines such as monocyte chemoattractant protein-1, TNF-α, IL-1β and IL-6.^93^ Similarly, in a rat model of cerebral ischemia, O_3_ inactivated the NF-κB signaling pathway, reducing TLR4 levels and, in turn, inflammation.^94^ O_3_ inhibited TLR4/NF-κB signaling in doxorubicin-treated rat cardiomyoblasts *in vitro*; as a consequence, the expression of IL-1β, IL-6, TNF-α, matrix metalloproteinase (MMP)-2, and MMP-9 decreased.^95^ A similar effect was achieved in a human melanoma cell line, in which O_3_ treatment induced an anti-inflammatory response by reducing the activation of NF-κB/p65, with the consequent downregulation of different ILs (IL-1, IL-6, IL-8, IL-9, IL-17, IL-19, TNF-α, transforming growth factor (TGF)-β, vascular endothelial growth factor (VEGF), MMP-2, and MMP-9).^96^ In a rat model of renal transplantation, O_3_ treatment attenuated the expression of NF-κB/p65 in renal tissue^97^; this effect was associated with a subsequent reduction in the protein expression of high-mobility group box-1 (a transcription regulator involved in the inflammatory cascade). In parallel, in transplanted animals treated with O_3_, the systemic levels of IL-6, IL-18, and cyclooxygenase-2 (which generates immunosuppressive prostaglandins) decreased.^97^ Reduced inflammation through the modulation of NF-κB/p65 has also been reported in a rat endotoxic shock model after O_3_ treatment.^98^ Similarly, O_3_ exerted an anti-inflammatory effect by suppressing the expression of the NF-κB, TNF-α, and B-cell lymphoma 2 (Bcl-2)-associated X protein (Bax) genes in human lung alveolar cells.^99^

O_3_ exerts its anti-inflammatory effect through the modulation of other pathways, such as adenosine monophosphate-activated protein kinase (AMPK)/class A1 scavenger receptors. Specifically, O_3_ activates AMPK, which in turn enhances class A1 scavenger receptor 1 expression and function, leading to an increase in the ability of macrophages to engulf and clear neutrophil extracellular traps from inflamed tissue, as demonstrated in mouse sepsis-induced acute lung injury.^100^

Another pathway involved in the anti-inflammatory effect of O_3_ depends on the inflammation-related transcription factor signal transducer and activator of transcription 3 (STAT3). In detail, the effects of O_3_ on the STAT3 and extracellular signal-regulated kinase pathways have been investigated in keratinocytes in atopic dermatitis, where IL-4 activates STAT3 to promote the transcription of thymic stromal lymphopoietin (a cytokine that regulates T-cell activity) and inhibit filaggrin expression.^101^ Moreover, O_3_ downregulates the phosphorylation of the mitogen-activated protein kinase family member p38, which regulates the pro-inflammatory cytokines IL-1β and TNF-α and c-Jun N-terminal kinase (responsible for STAT3 phosphorylation).^101^ O_3_ also protected rat cardiomyocytes from ischemia–reperfusion injury by activating the Janus kinase 2/STAT3 pathway, which in turn upregulated heat shock protein 70 expression.^102^

O_3_ treatment has been shown to exert anti-inflammatory effects by modulating or inhibiting the activation of inflammatory caspases and inflammasomes. In a rat model of chronic kidney disease, following O_3_ treatment, the levels of IL-1β were correlated with the decreased expression of NLRP3, a multi-protein intracellular complex that plays a crucial role in the innate immune response.^103^ In a rat model of myocardial ischemia-reperfusion, O_3_ reduced injury by decreasing the expression of NLRP3, pro-caspase-1, and ASC, as well as the secretion of caspase-1, IL-1β, and IL-18.^104^ In a mouse model of acute lung injury, O_3_ downregulated the expression levels of the proteins NLRP3, ASC, caspase-1, and caspase-1 p20 in lung tissue and reduced the levels of myeloperoxidase (MPO), inflammatory cells, and the pro-inflammatory cytokines TNF-α, IL-6, IL-1β, and IL-18.^105^ Similarly, ozonated oil applied *in vivo* on a mouse model of sepsis and *in vitro* on cultured macrophages and monocytic leukemia cells was able to suppress the activation of the NLRP3 inflammasome, thus leading to the inhibition of caspase-1 cleavage and a reduction in IL-1β and IL-18 levels.^106^ In a rat nephropathy model, O_3_ reduced the expression of caspase-1, -3 and -9, hypoxia-inducible factor-1α (which promotes cell survival under hypoxic conditions), and *TNF-α* genes.^107^

In addition to modulating the previously mentioned pathways, O_3_ exerts its anti-inflammatory effects through the activation of the Nrf2 pathway. In fact, the application of ozonated oil could increase the expression of Nrf2 and reduce the expression of several pro-inflammatory factors, such as IL-1β and TNF-α, NOS2 and MMP-2 in a model of human colonic epithelial and lipopolysaccharide-stimulated monocytic cells.^108^ Similarly, ozonated saline administered *in vitro* to murine microglial and human endothelial cells has been proved to upregulate *Nrf2* and *SOD1* genes, reduce the pro-inflammatory markers iNOS and IL-1β, and increase the anti-inflammatory markers, arginase-1 and IL-10.^109^ *In vitro* studies on cell lines, such as human fibroblasts^110^ and microglia,^111^ demonstrated that O_3_ uniquely influenced the release of IL-6, TNF-α, and TGF-β1 in cells that had been lipopolysaccharide-activated compared with non-activated cells; this effect was linked to differences in Nrf2 activation in the two cell populations. The anti-inflammatory effect of O_3_ through the modulation of Nrf2 has also been demonstrated in a mouse model of atopic dermatitis^101^ and in rats with lung ischemia–reperfusion injury.^40^

### Anti-inflammatory response in blood cells

O_3_ treatment induces the release of interferon (IFN)-γ^112^ and TNF-α^113^ and inhibits the release of IL-4, IL-6 and IL-10^114^ in human leukocytes. Changes in cytokines, ILs and other factors after O_3_ treatment were also observed in human platelets, in association with the release of platelet-derived growth factor, TGF-β1 (which is involved in cell proliferation, differentiation, and apoptosis) and IL-8.^115^ Furthermore, the application of O_3_ to human platelet-rich plasma (PRP) significantly increased the release of IL-2 and IFN-α, along with the secretion of growth factors, such as epidermal growth factor, fibroblast growth factor 2 (which is involved in cell growth, morphogenesis, and tissue repair), and VEGF (which plays pivotal roles in vasculogenesis and angiogenesis).^116^ Blood ozonation also induced a transient increase in TNF-α in rabbits *in vivo*.^117^ Treating cultured human endothelial cells with ozonated human serum stimulated the release of IL-8 while inhibiting E-selectin, a protein found on inflamed endothelial cells.^118^

### Anti-inflammatory response in animal models of pathological conditions

O_3_ was found to be crucial for maintaining immune balance in cultured human colon cancer cells, where TNF-α, MMP-2 and MMP-9 (which are involved in cytokine activation) were upregulated, whereas IL-1β, IL-6 and IL-8 were downregulated.^119^ However, the bulk of evidence for the O_3_-driven modulation of inflammatory mediators was obtained in animal models of pathological conditions (e.g., dyslipidemia, hepatic inflammation and steatosis, diabetic nephropathy and retinopathy, rheumatoid arthritis, septic lung injury, acute distal colitis, intestinal sepsis, postoperative uterine adhesion formation, pelvic inflammatory disease, mammary gland inflammation, uveitis, atopic dermatitis, demyelination) or ischemia–reperfusion injury; here, the levels of pro-inflammatory cytokines (e.g., IL-1β, IL-2, TNF-α) were reduced, and those of anti-inflammatory ILs (e.g., IL-10) increased.^107^,^120^,^121^,^122^,^123^,^124^,^125^,^126^,^127^,^128^,^129^,^130^,^131^,^132^,^133^,^134^,^135^,^136^,^137^

In zebrafish subjected to caudal fin resection, O_3_ modulated the expression of IL-6, IL-10, IL-1β, and TNF-α, thus promoting tissue regeneration.^85^,^138^ Similarly, O_3_ improved wound healing in rabbits^139^ and in a rat peritonitis model^140^ and in a rat radiation dermatitis model^141^ by decreasing the levels of TNF-α and IL-6 and increasing the expression of TGF-β and platelet-derived growth factor. O_3_ also improved the healing of skin ulcers in rats by reducing the levels of MDA and TNF-α.^142^ In addition to promoting wound healing, O_3_ improved the structural and functional recovery of spine injury by reducing the levels of IL-6,^143^ TNF-α, IL-1α, and complement component 1q.^84^ The O_3_-driven anti-inflammatory response also enhanced the action of wound healing-promoting drugs; for instance, ozonated oil applied to skin leishmaniasis lesions exerted leishmanicidal activity and improved healing by decreasing TNF-α levels and lowering blood leukocytes.^144^ Finally, O_3_-rich nanocomposite hydrogels counteracted cartilage destruction due to synovial inflammation by reducing the levels of IL-1β, IL-6 and TNF-α.^145^

### Anti-inflammatory response in pain managenment

The ability of O_3_ to modulate cytokine production significantly contributes to pain-relieving properties. *In vitro* and *in vivo* studies performed on a rat model of intervertebral disc and chronic radiculitis demonstrated that O_3_ administration downregulated spinal TNF-α, IL-1β and IL-6 levels, thus alleviating pain.^146^,^147^ O_3_ also exerts its anti-inflammatory effects on pain by influencing different pathways that are critical to both pain perception and chronicity. For instance, O_3_ was found to modulate pain through the phosphodiesterase 2A-cyclic guanosine monophosphate/cyclic adenosine monophosphate-NF-κB/p65 signaling pathway.^147^ In murine models of sciatic nerve injury and gout, O_3_ reduced inflammation and pain by activating AMPK and upregulating the Gas6/MerTK/SOCS3 signaling pathway (the growth arrest-specific-6 protein, Gas6 is involved in cell proliferation, while MER proto-oncogene tyrosine kinase, *MerTK* codes for receptors promoting macrophage activity, and the suppressor of cytokine signaling 3, SOCS3, regulates cytokine or hormone signaling to control inflammation homeostasis).^148^,^149^ Similarly, O_3_-driven upregulation of the AMPK-SOCS3 pathway alleviated neuropathic pain in a mouse model of peripheral neuropathy.^150^ Specifically, O_3_ activated AMPK and increased the expression of SOCS3, which in turn reduced the levels of tissue factor (involved in blood coagulation), c-Fos (a protein crucial for cell proliferation, differentiation, and survival), and calcitonin gene-related peptide (a potent vasodilator linked to pain) while suppressing microglial activation. Moreover, in a chronic constriction injury model of sciatic nerves in rats, O_3_ administration reduced neuropathic pain through its effects on the spinal glutamate receptor 6-NF-κB/p65 signaling pathway.^151^ Finally, subcutaneous O_3_ injections decreased mechanical allodynia in a mouse model of neuropathic pain^152^ by reducing the expression of *caspase-1*, *-8*, and *-12* genes and the levels of IL-1β in the orbito-frontal cortex.

Methodological details of the experimental investigations reviewed in this chapter are reported in **Additional Table 3**. **Additional Table 4** summarizes the molecules involved in the anti-inflammatory properties of low-concentration O_3_, and **Figure 2** shows a schematic representation of the recognized molecular pathways responsible for the anti-inflammatory effects of O_3_.

**Additional Table 3:** The experimental investigations on the cellular and molecular mechanisms for anti-inflammatory effect of low O_3_ concentration

Experimental model	Target organ/tissue	O_3_ treatment	Administration route	Reference
***In vitro***				
Human blood mononuclear cells		1–80 µg O_3_/mL O_2_; Single treatment	Medium	1
Human platelets		20–80 µg O_3_/mL O_2_; Single treatment	Medium	2
Human platelet rich plasma		16 µg O_3_/mL O_2_; Single treatment	Medium	3
HUVECs		20–80 µg O_3_/mL O_2_; Single treatment	Medium	4
Human HT29 colon cancer cells		10–50 µg O_3_/mL O_2_; Single treatment	Medium	5
Human CHL-1 melanoma cancer cells		10–50 µg O_3_/mL O_2_; Single treatment	Medium	6
Human lung alveolar cells		20 μM; Single treatment	Medium	7
Human HT29 colon cancer cells Human THP-1 monocytic cells		0.1–2 mg/mL; Single treatment	Medium	8
Human fibroblasts		10–30 μg O_3_/mL O_2_; Single treatment	Medium	9
HMCs		10, 20 μg O_3_/mL O_2_; Single treatment	Medium	10
HUVECs Mouse BV-2 microglia		1–10 µg O_3_/mL O_2_; Single treatment	Medium	11
***Ex vivo***				
Human healthy donors, rabbit	Blood	54 μg O_3_/mL O_2_; 30 and 200 mL	In syringe, in bag	12
***Ex vivo* and *In vitro***				
Human healthy donors; Human peripheral blood mononuclear cells	Blood	0.2–108 µg O_3_/mL O_2_; 4.5 mL.	Medium	13
Human healthy donors; Human peripheral blood mononuclear cells	Blood	2.2–108 µg O_3_/mL O_2_; 4.5 mL	Medium	14
***In vivo***				
Rat model of sepsis injury	Liver	20 µg O_3_/mL O_2_; 4 mL; Single treatment	Intraperitoneal	15
Rat radiation dermatitis model	Skin	300–1200 mg/mL; Twice a day for 7 d	Topical	16
Rat model of renal transplantation	Kidney	50 μg O_3_/mL O_2_; 1.1 mg/kg; Daily for 15 d	Rectal insufflation	17
Rat model of ischemia-reperfusion injury	Lung	100 µg O_3_/kg; 2 mL; Daily for 10 d	Intraperitoneal	18
Rat model of partial hepatectomy after ischemia-reperfusion injury	Liver	60 µg O_3_/mL O_2_; 1.2 mg/kg; Daily for 5 d	Intraperitoneal	19
Rat model of renal ischemia-reperfusion injury	Kidney	50 µg O_3_/mL O_2_; 1 mg/kg; Daily for 15 d	Rectal insufflation	20
Rat model of diabetic nephropathy	Kidney	50 µg O_3_/mL O_2_; 1.1 mg/kg; Daily for 6 wk	Intraperitoneal	21
Rat model of rheumatoid arthritis	Spleen	20 µg O_3_/mL O_2_; 80 mg/kg; 3 times for 3.5 wk	Intra-articular injection	22
Rat model of rheumatoid arthritis	Knee joint	35 mg O_3_/L O_2_; 1 to 3 times per wk	Intra-articular injection	23
Rat model of septic lung injury	Lung	60 mg O_3_/mL O_2_; 0.7 mg/kg; Daily for 5 d	Intraperitoneal, rectal insufflation	24
Rat model of acute distal colitis	Colon	25 µg O_3_/mL O_2_; 1 mg/kg; Daily for 3 wk	Intraperitoneal, rectal insufflation	25
Rat model of uterine adhesion	Uterus, peritoneal fluid	60 mg O_3_/mL O_2_; 0.7 mg/kg; Daily for 3 d	Intraperitoneal	26
Rat model of endometrial injury	Uterus	45, 60 µg O_3_/mL O_2_; 0.3 mL; 7 d	Intraperitoneal	27
Rat model of diabetic retinopathy	Retina	50 μg O_3_/mL O_2_; 0.5 mg/kg; 3 times per week for 1 mon	Rectal insufflation	28
Rat model of experimental uveitis	Eye	60 mg O_3_/mL O_2_; 0.7 mg/kg; Daily for 1 wk	Intraperitoneal	29
Rat model of demyelination	Brain	25–50 μg O_3_/mL O_2_; 0.5–1 mg/kg; 5 times per week	Rectal insufflation	30
Rat model of skin ulcer	Skin	100 μg O_3_/mL O_2_; 0.5 mL; Twice a day for 14 d	Topical	31
Rat model of chronic kidney disease	Kidney	50 μg O_3_/mL O_2_; 1.1 mg/kg; Daily for 4 wk	Rectal insufflation	32
Rat model of cerebral ischemia	Brain	2 mg O_3_/kg; Single treatment.	Intraperitoneal	33
Rat model of endotoxic shock	Liver	2% O_3_; 0.5 mg/kg; Daily for 5 d	Intraperitoneal	34
Rat model of chronic kidney disease	Kidney	50 μg O_3_/mL O_2_; 1.1 mg/kg; Once a day for 2 wk	Rectal insufflation	35
Rat model of chronic radiculitis	Spinal Cord	10–30 μg O_3_/mL O_2_; 20 μL; Single treatment	Intrathecal	36
Rat model of sciatic nerve injury	Spinal cord	10–30 μg O_3_/mL O_2_; 20 μL; Single treatment	Intrathecal injection	37
Mouse model of sciatic nerve injury	Orbito-frontal cortex, hind paw	90, 180 μg O_3_/kg; Single administration	Subcutaneous	38
Mouse model of acute lung injury	Lung	10, 20 µg O_3_/mL O_2_; 1.1 mg/kg; Single treatment	Rectal insufflation	39
Mouse model of acute lung injury	Lung	10, 20 µg O_3_/mL O_2_; 1.1 mg/kg; Single treatment	Rectal insufflation	40
Mouse model of acute lung injury	Lung	15 μg O_3_/mL O_2_; 0.5 mL; Single treatment	Intraperitoneal	41
Mouse model of atopic dermatitis	Skin	0.1 mL O_3_/cm^2^; Daily for 1 wk	Topical	42
Mouse model of obesity	Liver	0.5% O_3_; 4 wk	Oral	43
Rabbit model of wound healing	Skin	70 μg O_3_/mL O_2_; 12 mL; 6 treatments every other day	Rectal insufflation	44
Rabbit model of peritonitis	Colon	25 μg O_3_/mL O_2_; 1 mg/kg; Daily for 3 wk	Intraperitoneal	45
Goat model of inflamed mammary glands	Mammary gland	2000 ppm O_3_; 0.1 mL; Single treatment	Intramammary infusion	46
Zebrafish model of fin regeneration	Caudal fin	0.2 mg/L; Ozonated water every 12 h	Water	47
***In vivo* and *in vitro***				
Rat model of spinal cord injury; Mouse RAW264.7 macrophage cells; Primary spinal cord neurons	Spinal cord	Once for 3 d	Reinfusion of ozonated blood	48
Rat model of myocardial ischemia–reperfusion injury; Rat cardiomyocytes	Hearth	5–80 μg O_3_/mL O_2_; 50, 100 μg/kg; Daily for 5 d	Intraperitoneal	35
Rat model of myocardial ischemia–reperfusion	Hearth	20–40 μg O_3_/mL O_2_; 1 mg/kg; Daily for 5 d	Rectal insufflation	49
Mouse model of cutaneous lesions; Peritoneal macrophages	Skin	0.625–320 μg O_3_/mL O_2_; 250 μL; Daily for 30 d	Topical Medium	50
Mouse model of sepsis; Mouse macrophages; Human THP-1 monocytic cells	Lung	0.736 g O_3_/mL O_2_; 0.5–200 μL; Single treatment	Intraperitoneal Medium	51
Mouse model of peripheral neuropathy; Mouse RAW 264.7 macrophages	Sciatic nerve, L4-L5 lumbar segments	7.5–30 μg O_3_/mL O_2_; 30 μg/mL solution; Daily for 7 d	Intraperitoneal Medium	52

HMC: Human microglial clone; HUVEC: human umbilical vein endothelial cell; O_3_: ozone.

**Additional Table 4:** Molecule modulation of low O_3_ concentration and accounting for anti-inflammatory effects

Experimental model	Molecules	Reference
***In vitro***		
Human leukocytes	TNF-α, IL-4, IL-6, IL-10	1 2 3
Human platelets	IFN-α, TGF-β1, IL-2, IL-8, EGF, FGF-2, PDGF, VEGF	4 5
Human endothelial cells	IL-8, E-selectin	6
Human colon cancer cells	TNF-α, MMP-2, MMP-9, IL-1β, IL-6, IL-8	7
Human melanoma cells	NF-κB/p65, IL-1, IL-6, IL-8, IL-9, IL-17, IL-19, TNF-α, TGF-β, VEGF, MMP-2, MMP-9	8
Human lung alveolar cells	NF-κB, TNF-α, Bax	9
Human colonic epithelial and monocytic cells	Nrf2, IL-1β, TNF-α, NOS2 and MMP-2	10
Human fibroblasts and microglia	Nrf2, IL-6, TNF-α, TGF-β1	11 12
Human endothelial cells and Mouse microglia	iNOS, IL-1β, arginase-1, IL-10	13
Rat cardiomyoblasts	TLR4, NF-κB, IL-1β, IL-6, TNF-α, MMP-2, MMP-9	14
Rat cardiomyocites	JAK2/STAT3, HSP70	15
***In vivo***		
Rat, mouse, rabbit, goat, zebrafish	C1q, IL-1α, IL-1β, IL-2, IL-6, IL-10, IL-12, IFN-γ, TNF-α	16 17 18 19 20 21 22 23 24 25 26 27 28 29 30 31 32 33 34 35 36 37 38 39 40 41 42 43 44
Rat	TLR4, p-NF-κB/p65, MCP-1, TNF-α, IL-1β, IL-6	45 46
Rat	p65/NF-κB, HMGB1, IL-6, IL-18, COX-2	47
Rat	NLRP3, IL-1β	48
Rat	caspase-1, caspase-3, caspase-9, HIF-1α, TNF-α	49
Rat	GluR6-NF-κB/p65	50
Rat, mouse	Nrf2	51 52
Mouse	AMPK/SR-A	53
Mouse	AMPK/Gas6/MerTK/SOCS3, tissue factor, c-Fos, CGRP	54 55 56
Mouse	caspase-1, caspase-8, caspase-12, IL-1β	57
***In vivo and in vitro***		
Rat, hamster ovary cells	PDE2A-cGMP/cAMP-NF-κB/p65, TNF-α, IL-1β, IL-6	58 59
Rat, mouse, mouse macrophages, human monocytic-leukemia cells	NLRP3, procaspase-1, ASC, caspase-1, IL-1β, IL-6, IL-18, TNF-α	60 61 62
Mouse, mouse macrophage cells	STAT3, ERK, IL-4, p38, IL-1β, TNF-α, p-JNK	52

AMPK: Adenosine monophosphate-activated protein kinase; ASC: apoptosis-associated speck-like protein containing a caspase recruitment domain; Bax: B-cell lymphoma 2-associated X protein; C1q: complement component 1q; cAMP: cyclic adenosine monophosphate; cGMP: cyclic guanosine monophosphate; CGRP: calcitonin gene-related peptide; COX-2: cyclooxygenase-2; EGF: epidermal growth factor; ERK: extracellular signal-regulated kinase; FGF-2: fibroblast growth factor 2; Gas6: growth arrest-specific-6 protein; GluR6: glutamate receptor 6; HIF-1α: hypoxia-inducible factor; HMGB1: high-mobility group box-1; HSP70: heat shock protein 70; IFN: interferon; IL: interleukin; iNOS: inducible nitric oxide synthase; JAK2: Janus kinase 2; MCP-1: monocyte chemoattractant protein-1; MerTK: MER proto-oncogene tyrosine kinase; MMP: matrix metalloproteinase; NF-κB: nuclear factor kappa-light-chain-enhancer of activated B cells; NLRP3: nucleotide-binding oligomerization domain-like receptor family pyrin domain containing 3; NOS: nitric oxide synthase; Nrf2: nuclear factor erythroid 2-related factor 2; O3: ozone; PDE2A: phosphodiesterase 2A; PDGF: platelet-derived growth factor; p-JNK: phosphorylated c-Jun N-terminal kinase; p-NF-κB: phosphorylated nuclear factor kappa-light-chain-enhancer of activated B cells; SOCS3: suppressor of cytokine signaling 3; SR-A: class A scavenger receptor; STAT3: signal transducer and activator of transcription 3; TGF: transforming growth factor; TLR4: Toll-like receptor 4; TNF-α: tumor necrosis factor α; VEGF: vascular endothelial growth factor.

**Figure 2:**
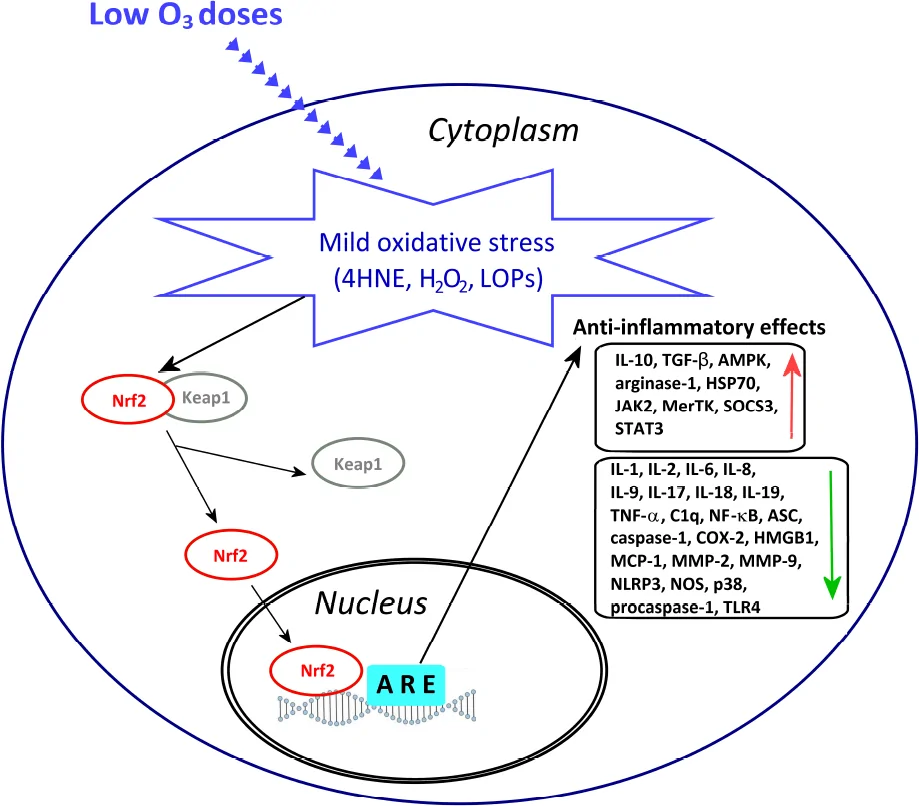
The molecular pathways involved in the anti-inflammatory effects of low-concentration O_3_. Created with Jasc Paint Shop Pro (version 7.04). 4-HNE: 4-Hydroxynonenal; AMPK: adenosine monophosphate-activated protein kinase; ARE: antioxidant response element-driven genes; ASC: apoptosis-associated speck-like protein containing a caspase recruitment domain; C1q: complement component 1q; COX-2: cyclooxygenase-2; H_2_O_2_: hydrogen peroxide; HMGB1: high-mobility group box-1; HSP70: heat shock protein 70; IL: interleukin; JAK2: Janus kinase 2; Keap1: Kelch-like ECH associated protein 1; LOP: lipid oxidation product; MCP-1: monocyte chemoattractant protein-1; MerTK: MER proto-oncogene tyrosine kinase; MMP: matrix metalloproteinase; NF-κB: nuclear factor kappa-light-chain-enhancer of activated B cells; NLRP3: nucleotide-binding oligomerization domain-like receptor family pyrin domain containing 3; NOS: nitric oxide synthase; Nrf2: nuclear factor erythroid 2-related factor 2; O3: ozone; SOCS3: suppressor of cytokine signaling 3; STAT3: signal transducer and activator of transcription 3; TGF-β: transforming growth factor β; TLR4: Toll-like receptor 4; TNF-α: tumor necrosis factor α.

## Other Cellular Processes and Molecular Pathways Induced by Low-Concentration Ozone

The beneficial effects of low-concentration O_3_ mostly rely on antioxidant and anti-inflammatory mechanisms triggered in the organism by the gas oxidizing power. However, other cellular processes and molecular pathways may also be induced in response to O_3_-elicited eustress.

### Apoptosis and autophagy

Low-concentration O_3_ has been proved to modulate apoptotic and autophagic processes in various experimental models that mimic pathological conditions.

A study in a rat model of lung ischemia–reperfusion injury demonstrated that O_3_ was able to reduce the apoptotic rate through ASC, pro-caspase-1 and caspase-1,^40^ whereas in kidney ischemia–reperfusion, O_3_ prevented mitogen-activated protein kinase activation, thus reducing apoptosis in both *in vivo* and *in vitro* models and ameliorating organ preservation.^46^ A reduction in the apoptotic rate was also observed in a renal transplantation rat model^38^ due to the activation of the Nrf2/HO-1 signaling pathway, and in an ischemia–reperfusion rat model,^40^,^153^ where O_3_ downregulated TGF-β1, phospho-small mother against decapentaplegic 2 protein (a signal transducer for TGF-β receptors), NLRP3, ASC, procaspase-1, and caspase-1. O_3_ reduced apoptosis in a rat model of spinal cord injury and improved post-surgical recovery by decreasing oxidative stress and inflammation.^143^ In a mouse model of chronic constriction injury of the sciatic nerve, O_3_ reduced apoptosis by activating AMPK and MerTK, upregulated Gas6 and enhanced the phagocytic activity of bone marrow-derived macrophages against apoptotic neutrophils. In addition, O_3_ promoted the expression of SOCS3 and suppressed the expression of IL-1β, IL-6, and TNF-α.^154^

O_3_ inhibited autophagy in a rat model of cerebral ischemia by decreasing the expression of Beclin-1 (a regulator of autophagic cell death) and microtubule-associated protein 1A/1B-light chain 3 (LC3) (essential for autophagosome formation) but increasing the expression of p62 (which, in contrast, is known to decrease following autophagy induction).^94^ Similarly, O_3_ was found to reduce stroke-induced brain damage by inhibiting the key protein of autophagy, Bcl-2, in astrocytes both in oxygen–glucose deprivation/reoxygenation-induced human glioblastoma cells and in a middle cerebral artery occlusion rat model.^155^ In an *in vitro* model of oxygen–glucose deprivation/reperfusion in the myocardium, O_3_ inhibited the autophagy pathway by maintaining the proper Bcl-2/Bax ratio and normal levels of cleaved caspase-3, reducing the levels of Beclin-1 and autophagy protein 5 (which are needed for autophagosome formation), and decreasing the LC3-II/LC3-I ratio.^156^ In a rat model of radiculoneuritis, the expression of LC3, Beclin-1 and caspase-3 decreased after O_3_ treatment, resulting in the inhibition of autophagy and apoptosis in nerve root cells, with reduced nerve degeneration and pain.^157^ The intra-articular injection of O_3_ increased the levels of collagen-2 and LC3-II in a rat model of osteoarthritis and reduced the expression of p62 and MMP-13 (an enzyme with potent proteolytic capability that is especially expressed in osteoarthritis and rheumatoid arthritis). These findings suggest that increased autophagy in chondrocytes may protect against stress.^158^ In an *in vitro* model of osteoarthritis, O_3_ increased autophagy in primary chondrocytes of rats by activating the peroxisome proliferator-activated receptor γ/mechanistic target of rapamycin pathway.^159^

Methodological details of the experimental investigations reviewed are reported in **Additional Table 5**. **Additional Table 6** summarizes the molecules involved in the modulation of apoptosis and autophagy by low-concentration O_3_.

**Additional Table 5:** The experimental investigations on the cellular and molecular mechanisms for the effects of low O_3_ concentration on apoptosis and autophagy

Experimental model	Target organ/tissue	O_3_ treatment	Administration route	Reference
***In vitro***				
Rat H9C2 cardiomyoblasts		10–60 µg O_3_/mL O_2_; Single treatment	Medium	1
Rat primary chondrocytes		30–70 µg O_3_/mL O_2_; Single treatment	Medium	2
***In vivo***				
Rat model of lung ischemia/reperfusion injury	Lung	100 µg O_3_/kg; 2 mL; Daily for 10 d	Intraperitoneal	3
Rat model of renal ischemia/reperfusion injury	Kidney	50 µg O_3_/mL O_2_; 1–2 mg/kg; Daily for 15 d	Rectal insufflation	4
Rat model of cerebral ischemia	Brain	2 mg O_3_/kg; Single injection	Intraperitoneal	5
Rat model of kidney transplantation	Kidney	50 µg O_3_/mL O_2_; 1 mg/kg; Daily for 15 d	Rectal insufflation	6
Rat model of ischemia/reperfusion	Kidney	50 mg O_3_/L O_2_; 0.5 mg/kg; Daily for 10 d	Rectal insufflation	7
Rat model of radiculoneuritis	Nerve	30μg O_3_/mL O_2_; 50 μL; Daily for 3 d	Epidural	8
Rat model of osteoarthritis	Knee joint	30 µg O_3_/mL O_2_; 0.5 mL; 3 treatments	Intra-articular	9
***In vivo* and *in vitro***				
Rat model of cerebral artery occlusion. Human A172 glioblastoma cells	Brain	20–150 µg O_3_/mL O_2_	Medium	10
Mouse model of chronic sciatic nerve injury Mouse bone marrow-derived macrophages	Sciatic nerve	15–45 µg O_3_/mL O_2_; 30 μg O_3_/mL O_2_; Daily for 7 d	Intraperitoneal or medium	11

O_3_: Ozone.

**Additional Table 6:** Molecules modulation of low O_3_ concentration on apoptosis and autophagy

Experimental model	Molecules	Reference
***In vitro***		
Rat chondrocytes	PPARγ/mTOR	1
Rat cardiomyoblasts	Bcl-2/Bax, caspase-3, Beclin-1, Atg5, LC3-II/LC3-I	2
***In vivo***		
Rat	ASC, procaspase-1, caspase-1	3
Rat	Nrf2/HO-1, SMAD 2, NLRP3, ASC, procaspase-1, caspase-1	3 4 5
Rat	Beclin-1, LC3, p62	6
Rat	LC3, Beclin-1, caspase-3	7
Rat	LC3-II, p62, MMP-13, collagen-2	8
Mouse	AMPK, MerTK, Gas6, SOCS3, IL-1β, IL-6, TNF-α	9
***In vivo* and *in vitro***		
Rat, rat kidney epithelial cells	MAPK	10
Rat, human glioblastoma cells	Bcl-2	11

AMPK: Adenosine monophosphate-activated protein kinase; ASC: apoptosis-associated speck-like protein containing a caspase recruitment domain; Atg5: autophagy protein 5; Bax: B-cell lymphoma 2-associated X protein; Bcl-2: B-cell lymphoma 2; Gas6: growth arrest-specific-6 protein; HO-1: heme oxygenase-1; IL: interleukin; LC3: microtubule-associated protein 1A/1B-light chain 3; MerTK: MER proto-oncogene tyrosine kinase; MMP: matrix metalloproteinase; mTOR: mechanistic target of rapamycin; NLRP3: nucleotide-binding oligomerization domain-like receptor family pyrin domain containing 3; Nrf2: nuclear factor erythroid 2-related factor 2; O3: ozone; PPARγ: peroxisome proliferator-activated receptor γ; SOCS3: suppressor of cytokine signaling 3; TNF-α: tumor necrosis factor α.

### Pro-regeneration

The regenerative properties of O_3_ are not only due to its antioxidant and anti-inflammatory effects, as already recalled in the previous chapters, but may also depend on the activation of other cellular and molecular responses. Treatment with low-concentration O_3_ may contribute to regenerative processes in different organs (e.g., skin,^160^,^161^,^162^,^163^,^164^,^165^ bone,^166^,^167^,^168^ and nerve^169^,^170^,^171^). This is also observed when O_3_ is administered in combination with other therapies, such as drugs,^172^,^173^ hyperbaric oxygen^174^,^175^ or PRP.^175^,^176^ Identifying the pathways responsible for these effects may therefore open promising prospects for the application of O_3_ in regenerative medicine.

As demonstrated by the results of the following experimental works, O_3_ stimulates fibroblasts, which are present in the connective tissues of all organs. Low-concentration O_3_ increases the migration ability of fibroblasts *in vitro*, improving wound healing.^177^ Accordingly, in an *in vivo* and *in vitro* study,^178^ ozonated oil ameliorated wound healing by increasing fibroblast migration and upregulating the expression of collagen I, α-smooth muscle actin, and TGF-β1 mRNA as well as the expression of fibronectin (a matrix glycoprotein that plays a major role in cell adhesion, proliferation, motility and differentiation), vimentin (an intermediate cytoskeletal filament), N-cadherin (a transmembrane protein involved in cell–cell adhesion), MMP-2, MMP-9, and the modulators of the insulin-like growth factor signaling, insulin-like growth factor-binding protein-3, 5, and 6. O_3_ also decreased the expression of the epithelial marker E-cadherin and the cellular senescence marker p16. O_3_ also promotes the epithelial–mesenchymal transition by increasing the phosphorylation of phosphoinositide 3-kinases, protein kinase B, and mechanistic target of rapamycin. O_3_ modulates the proliferation of human fibroblasts and activates the formation of surface protrusions, consequently promoting their motility.^110^ Notably, these effects depend on the fibroblast activation state, as O_3_ was able to stimulate non-activated fibroblasts but was ineffective at activated fibroblasts; this differential fibroblast responsiveness to O_3_ would be beneficial in the different phases of wound healing, since overactivation of fibroblasts would be excluded, thus reducing the possibility of keloid formation. Wound healing in rats treated with O_3_ was accompanied by an increase in fibroblast growth factor 2,^179^ suggesting that this factor may contribute to accelerating the repair process. Cutaneous wound healing in mice was accelerated by ozonated oil treatment, which increased the expression of proteins involved in the regulation of cell proliferation, namely, VEGF and the proliferating cell nuclear antigen (a cofactor of DNA polymerase delta in DNA replication and repair).^180^ Accordingly, increased levels of proliferating cell nuclear antigen were detected during cell wound closure in a human keratinocyte line treated with ozonated saline.^181^ The same beneficial effect of ozonated oil was observed during cutaneous wound healing in guinea pigs, where collagen deposition and fibroblast proliferation were increased.^182^ This O_3_-driven fibroblast activation paralleled the increased expression of platelet-derived growth factor, TGF-β, and VEGF.

O_3_ also promotes tissue regeneration by promoting angiogenesis, cell proliferation, and collagen synthesis. O_3_ improved colon healing in a rat peritonitis model^140^ by restoring normal levels of MDA, MPO (highly expressed in neutrophils), VEGF, and the antigen Kiel 67 (a nuclear factor expressed in proliferating cells). Similarly, in the colonic tissue of rats treated with O_3_ before radiotherapy, O_3_ increased the level of the main component of collagen, hydroxyproline, and the level of SOD, whereas compared with those in the untreated controls, the MDA level decreased.^183^

Some experimental findings have indicated that the ability of low-concentration O_3_ to balance catabolic and anabolic processes contributes to regeneration. In a rat model of intestinal ischemia–reperfusion, O_3_ reduced injury and improved recovery by increasing the expression of pro-proliferative factors and phosphorylated extracellular signal-regulated kinases and decreasing the expression of caspase-3.^184^ In a rabbit model of osteoarthritis, the combination of O_3_ and human PRP treatment increased the expression of type II collagen while decreasing the levels of MMP-1 and bone morphogenetic protein 2 (a factor promoting matrix turnover and repair), suggesting the restoration of the homeostasis between anabolic and catabolic activities in the cartilage extracellular matrix.^185^ Accordingly, in a rat model of osteoarthritis, both O_3_ and O_3_ combined with hyaluronic acid promoted type II collagen expression.^186^ The positive effects of combined treatment with O_3_ and PRP on tissue regeneration probably rely on the ability of O_3_ to induce the release of several platelet factors, such as epidermal growth factor, fibroblast growth factor 2, and VEGF,^116^ which in turn stimulate the activity of various cell types.

Osteoarthritis was mitigated by O_3_ treatment in association with exercise or intra-articular injection of mesenchymal stem cells, due to the increased expression of sex-determining region Y box 9 (which is involved in chondrocyte differentiation) and hypoxia-inducible factor-1.^187^ In an *in vitro* and *in vivo* study, O_3_-rich nanocomposite hydrogel acted as a protector from osteoarthritis by promoting chondrocyte proliferation, increasing the expression of collagen II and aggrecan in the cartilage matrix, and decreasing the levels of VEGF.^145^

O_3_ treatment improved bone regeneration in a rat model of bone defects by stimulating osteogenic activity, osteogenic differentiation and angiogenesis.^188^ Similarly, in a rat model of osteonecrosis, O_3_ treatment stimulated intra-trabecular mesenchymal cells and the formation of CD31-positive vasculature.^189^ Neovascularization was promoted after O_3_ administration in a rat skin flap model, in which the expression of VEGF and CD34 was increased.^190^

Oxidative stress and Nrf2 are known to play a primary role in regulating adipogenesis and adipocyte metabolism.^191^,^192^ The potential of O_3_ treatment in modulating white adipose tissue was therefore investigated in *in vitro* and *ex vivo* models. Low-concentration O_3_ has been proved to exert a pro-adipogenic effect by improving the differentiation of adipose stem cells^193^ while maintaining the lipid content in adult adipocytes by increasing Nrf2 protein levels and *HO-1* gene expression.^88^

Methodological details of the experimental investigations reviewed in this chapter are reported in **Additional Table 7**. **Additional Table 8** summarizes the molecules involved in the pro-regenerative properties of low-concentration O_3_.

**Additional Table 7:** The experimental investigations on the cellular and molecular mechanisms for the pro-regenerative effects of low O_3_ concentration

Experimental model	Target organ/tissue	O_3_ treatment	Administration route	Reference
***In vitro***				
Human platelet rich plasma		16 µg O_3_/mL O_2_; Single treatment	Medium	1
Human fibroblasts		10–30 μg O_3_/mL O_2_; Single treatment	Medium	2
Human HaCaT keratinocytes.		2–300 µM O_3_; Single treatment	Medium	3
Human adipose-derived adult stem cells		5–20 µg O_3_/mL O_2_; Once at days 6, 16 and 20	Medium	4
Human HaCaT keratinocytes. Mouse L929 fibroblasts		0.25–8 µg O_3_/mL O_2_; 2 treatments	Medium	5
***Ex vivo***				
Mouse	Adipose tissue	10-100 µg O_3_/mL O_2_; Single treatment	Medium	6
***In vivo***				
Rat model of skin injury	Skin	3000 µg O_3_/L O_2_; Daily	Topical Subcutaneous	7
Rat model of sciatic nerve injury	Sciatic nerve	30 µg O_3_/mL O_2_; 1 mL; 0.7 mg/kg; Daily for 14 d	Perineural injection Intraperitoneal	8
Rat model of knee osteoarthritis	Knee joint	30 µg O_3_/mL O_2_; 0.015 mL, 0.2 mL; 2 mL; Once a week for 5 wk	Intra-articular Intraperitoneal	9
Rat model of colon anastomosis	Colon	60 mg O_3_/mL O_2_; 1.2 mg/kg; Daily for 5 d	Intraperitoneal	10
Rat model of intestinal ischemia–reperfusion injury	Small intestine	0.7 mg/kg; Daily	Intraperitoneal Intraluminal	11
Rat model of bone defect	Skull bone	0.7 mL O_3_/kg; Every 2 d	Intraperitoneal	12
Rat model of osteonecrosis	Femur	30 µg O_3_/mL O_2_; 1 mL; Every other day for 10 wk	Intraperitoneal	13
Mouse model of wound healing	Skin	8 µL ozonated oil; Twice a day up to 14 d	Topical	14
Rabbit model of peritonitis	Colon	25 μg O_3_/mL O_2_; 1 mg/kg; Daily for 3 wk	Intraperitoneal	15
Rabbit model of osteoarthritis	Knee joint	20 µg O_3_/mL O_2_; 40 µg O_3_ per treatment; Once a week for 6 wk	Intra-articular	16
Pig model of wound healing	Skin	0.1 mL ozonated oil; Daily	Topical	17
***In vivo* and *in vitro***				
Mouse model of wound healing; Mouse fibroblasts	Skin	400 µL ozonated oil; Once every 2 d for 12 d; 10 µL ozonated oil per well; Single treatment	Topical Medium	18

O_3_: Ozone.

**Additional Table 8:** Molecule modulation of low O_3_ concentration for regenerative effects

Experimental model	Molecules	Reference
***In vitro***		
Human keratinocytes	PCNA	1
***Ex vivo***		
Mouse explanted adipose tissue	Nrf2, HO-1	2
***In vivo***		
Rat	FGF-2	3
Rat	MDA, MPO, VEGF, Ki-67	4
Rat	hydroxyproline, MDA, SOD	5
Rat	p-ERK, caspase-3	6
Rat	Type II collagen	7
Rat	SOX9, HIF-1	8
Rat	VEGF, CD34	9
Mouse	VEGF, PCNA	10
Rabbit	Type II collagen, MMP-1, BMP-2	11
Guinea pig	PDGF, TGF-β, VEGF	12
***In vivo* and *in vitro***		
Mouse, mouse fibroblasts	Collagen I, α-SMA, TGF-β1 mRNA, fibronectin, vimentin, N-cadherin, MMP-2, MMP-9, IGFBP-3, IGFBP-5, IGFBP-6, E-cadherin, p16, PI3K, Akt, mTOR	13
Mouse, mouse macrophages and chondrocytes	Collagen II, aggrecan, VEGF	14

Akt: Protein kinase B; BMP-2: bone morphogenetic protein 2; FGF-2: fibroblast growth factor 2; HIF-1: hypoxia-inducible factor 1; HO-1: heme oxygenase-1; Ki-67: antigen Kiel 67; IGFBP: insulin-like growth factor-binding protein; MDA: malondialdehyde; MMP: matrix metalloproteinase; MPO: myeloperoxidase; mTOR: mechanistic target of rapamycin; Nrf2: nuclear factor erythroid 2-related factor 2; p-ERK: phosphorylated extracellular signal-regulated kinase; PCNA: proliferating cell nuclear antigen; PDGF: platelet-derived growth factor; PI3K: phosphoinositide 3-kinase; SOD: superoxide dismutase; SOX9: sex-determining region Y box 9; TGF: transforming growth factor; VEGF: vascular endothelial growth factor; α-SMA: α-smooth muscle actin.

### Anticancer

The application of O_3_ as an anticancer agent is controversial, especially because of the role of Nrf2, which is involved in cancer initiation and progression and may exert protective effects on tumor cells during chemotherapy.^194^,^195^ In fact, contradictory results have been reported: an *in vitro* study on a human breast cancer cell line reported interference between O_3_ and doxorubicin,^196^ whereas it also demonstrated that O_3_ did not affect the proliferation and migration of human cervical and breast cancer cells, nor reduced tamoxifen cytotoxicity through stimulation of the Nrf2 pathway.^197^,^198^

*In vitro* studies on different tumor cell lines from liver,^199^,^200^,^201^ breast,^202^ neuroblastoma,^203^ pancreas,^204^ lung,^205^ glial,^205^ and colon^206^ carcinoma have demonstrated that O_3_ can reduce cell proliferation, motility and invasion and induce cell cycle arrest and apoptotic cell death. Several mechanisms may account for these antitumor effects, namely, ROS accumulation,^199^,^201^ mitochondrial damage,^200^,^205^ the modulation of several proteins involved in the regulation of the cell cycle (e.g., p53, p21, cyclin D1, cyclin B1, cell division cycle-2, cyclin-dependent kinase (CDK)-1, CDK-4, CDK inhibitor 2A and Wee1)^199^,^203^,^204^ and apoptosis (e.g., cleaved poly(adenosine diphosphate ribose) polymerase-1, cytochrome c, caspase-3 and -9, Bax and phosphorylated c-Jun N-terminal kinase,^200^,^203^,^206^ and the pro-apoptotic genes p53, Cas9 and Cas3202), and decreases in the protein levels of Bcl-2, p65, NF-κB, STAT3, TNF-α, IL-6, Janus kinase 2 (a tyrosine kinase implicated in cytokine signaling), Slug and Twist (both transcription factors involved in cancer progression), vimentin, MMP-2, MMP-9 and high-mobility group box-1^200^,^201^ as well as the transcription factors E2F1 and E2F3.^204^ O_3_ also modulates gene expression, increasing the transcription of mRNAs encoding various pro-apoptotic genes^202^ and downregulating rat sarcoma virus (Ras)-associated pathway genes that are involved in cell proliferation, motility and chemoresistance of pancreatic ductal adenocarcinoma cells.^204^ When associated with antitumor drugs (e.g., gemcitabine and paclitaxel,^204^ doxorubicin,^207^ 5-fluorouracil and cisplatin^119^), O_3_ treatment enhances the antitumor effect by promoting apoptosis, increasing TNF-α, MMP-2 and MMP-9 levels, and decreasing IL-1β, IL-6 and IL-8 levels. Ozonated oil has an antiproliferative effect on human and murine melanoma cells (while leaving normal human keratinocytes undamaged) by upregulating the expression of ferroptosis-related genes, such as glutamate-cysteine ligase modifier subunit, HO-1, ferritin light chain, and sequestosome 1, and decreasing the expression of GSH and GPx4 proteins.^208^ O_3_ also acts as a radiosensitizer by increasing ROS production and DNA damage in melanoma and ovarian cancer cells under X-ray irradiation *in vitro*.^209^

The promising antitumor efficacy of O_3_ treatment has been demonstrated in *in vivo* studies in various animal models. In mice bearing hepatocellular carcinoma malignant ascites, O_3_ treatment reduced peritoneal fluid production and induced a decrease in the content of free DNA (i.e., the degraded DNA fragments released into body fluids in advanced cancer), IL-6, IFN-γ, MMP-9, VEGF, TNF-α, p-AMPK and class A scavenger receptor protein in the ascites, while reducing MPO and neutrophil extracellular trap-associated guanine histone H3 in the intestinal tissue.^210^ In a rabbit model of papillomavirus-associated auricular carcinoma, O_3_ exerted an antitumor effect by increasing the expression of genes encoding receptors essential for initiating the immune response (i.e., CD80/CD28, CD86/CD28, CD40/CD154 (CD40L), CD4, and CD1d) and increasing the levels of related downstream signaling molecules (e.g., the myeloid differentiation primary response gene 88-like, MyD88). In addition, O_3_ treatment upregulated the expression of IL-2, IL-10, IL-18 and IFN-γ and downregulated the expression of cyclooxygenase-2.^211^ O_3_ also demonstrated a preventive effect in a rat model of non-muscle invasive bladder cancer by increasing the antioxidant response of SOD.^212^

When used in association with radiotherapy, O_3_ treatment improved radiosensitivity in a mouse xenograft model of esophageal carcinoma,^213^ in a mouse model of peritoneal carcinomatosis^214^ and in a rat tongue cancer model.^215^ In fact, O_3_ has been shown to lower the peripheral blood levels of circulating free DNA, IFN-γ, MPO-DNA complexes, TNF-α, IL-6, hypoxia-inducible factor-1α and MMP-9 and to increase p-AMPK and class A scavenger receptor protein expression in tumor tissues.^213^

O_3_ showed antitumor effects even when it was loaded in nanoformulations. An ozonated nanoemulsion significantly reduced tumor recurrence in a mouse model of hepatocellular carcinoma by regulating the expression of several pro-apoptotic genes, such as GPx4, acyl coenzyme asynthetase long chain family member 4, and CDK inhibitor 1A.^216^ An oleogel loaded with O_3_ and doxorubicin was applied in combination with radiotherapy in a melanoma-bearing mouse model, inducing ROS accumulation, decreasing cell proliferation, increasing apoptosis and stimulating CD3^+^ and CD8^+^ T cells.^217^ After X-ray irradiation, a nanosystem consisting of liposomes enwrapping O_3_ and a molecule that acts as an inhibitor of CD47 (i.e., a cancer cell surface protein hampering macrophage phagocytosis) acted as a potent antitumor agent in both a mouse subcutaneous tumor model and a mouse mammary orthotopic tumor model, enhancing cytotoxic lymphocyte infiltration and reversing the immunosuppressive action of the tumor microenvironment.^218^ Similarly, a nanosystem consisting of a perfluorocarbon chain-modified cisplatin prodrug carrying O_3_ exerted antitumor effects after X-ray irradiation in both a mouse subcutaneous tumor model and a mouse lung metastatic tumor model because of the synergistic effect of cisplatin and hydroxyl radicals generated by O_3_ under irradiation.^219^

Methodological details of the experimental investigations reviewed in this chapter are reported in **Additional Table 9**. **Additional Table 10** summarizes the molecules involved in the anticancer properties of low-concentration O_3_.

**Additional Table 9:** The experimental investigations on the cellular and molecular mechanisms for the anti-cancer effects of low O_3_ concentration

Experimental model	Target organ/tissue	O_3_ treatment	Administration route	Reference
***In vitro***				
Human A549 lung adenocarcinoma cells Human U87MG glioblastoma cells		10% O_3_; Single treatment	Medium	1
Human Colo-320 and Colo-741 colon cancer cells		5–20 µg O_3_/mL O_2_; Single treatment	Medium	2
Human HT29 colon cancer cells		10–50 µg O_3_/mL O_2_; Single treatment	Medium	3
Human HeLa adenocarcinoma cells		10, 35 µg O_3_/mL O_2_; Single treatment	Medium	4
Human MCF-7 breast cancer cells		10, 20 µg O_3_/mL O_2_; Single treatment	Medium	5
Human Bel7402 and SMMC7721 liver cancer cells		5–30 µg O_3_/mL O_2_; Single treatment	Medium	6
Human BEL7402 hepatocellular carcinoma cells		2.07–1.5 µg O_3_/mL O_2_; Single treatment	Medium	7
Human MDA-MB-231 breast cancer cells; HUVECs		5–20 µg O_3_/mL O_2_; Single treatment	Medium	8
Human SK-N-SH and SK-N-DZ neuroblastoma cells		25 µg O_3_/mL O_2_; Treatments with a 1-hour break	Medium	9
Human A375, G361 and Malme-3M melanoma cells; Human HaCaT keratinocytes; Mouse B16F10 melanoma cells		0.01% O_3_; Single treatment or daily for 2 d	Medium	10
Human BT-474 breast cancer cells; Mouse L929 fibroblasts		30 µg O_3_/mL O_2_; Single treatment	Medium	11
Human MCF-7 breast cancer cells; Mouse L929 fibroblasts		30 µg O_3_/mL O_2_; Single treatment	Medium	12
***In vivo***				
Rat model of non-muscle invasive bladder cancer	Urinary bladder	25 µg/mL; 1 mL; 2–3 times per week	Intravesical instillation	13
Rat model of tongue cancer	Tongue	15 µg O_3_/mL O_2_; 1 mL; 4 treatments every 5 d	Rectal injection	14
Mouse model of esophageal carcinoma	Tumor tissue	30 µg O_3_/mL O_2_; 0.5 mL; Daily for 13 d	Intraperitoneal	15
Mouse model of hepatocellular carcinoma	Small intestine	15, 30 µg O_3_/mL O_2_; 0.5 mL; Daily for 13 d	Intraperitoneal	16
***In vivo* and *in vitro***				
Mouse model of hepatocellular carcinoma; Human BEL7402 hepatocellular carcinoma cells	Liver	5–40 µg O_3_/mL O_2_; 20 µg O_3_/mL O_2_; 0.2 mL, 10 mL; Daily for 10 d	Intratumor injection; Medium	17

O_3_: Ozone.

**Additional Table 10:** Molecule modulation of low O_3_ concentration for anti-cancer effect

Experimental model	Molecules	Reference
***In vitro***		
Human hepatocarcinoma cells	ROS, p53, p21, cyclin D1, cyclin B1, cdc2, CDK-1, CDK-4, CDKN2A, Wee1, PARP-1, cytochrome c, caspase-3, caspase-9, Bax, p-JNK, Bcl-2, p65, NF-κB, STAT3, TNF-α, IL-6, JAK2, Slug, Twist, vimentin, MMP-2, MMP-9, HMGB1	1 2 3 4 5
Human breast adenocarcinoma cells	p53	6
Human breast adenocarcinoma cells, human colon cancer cells	TNF-α, MMP-2, MMP-9, IL-1β, IL-6, IL-8	7 8
Human neuroblastoma cells	p53, p21, cyclin D1, cyclin B1, cdc2, CDK-1, CDK-4, CDKN2A, Wee1, PARP-1, cytochrome c, caspase-3, caspase-9, Bax, p-JNK	9
Human pancreatic adenocarcinoma cells	p53, p21, cyclin D1, cyclin B1, cdc2, CDK-1, CDK-4, CDKN2A, Wee1, E2F1, E2F3, TNF-α, MMP-2, MMP-9, IL-1β, IL-6, IL-8	10
Human and mouse melanoma cells	GSH, GPx4, ROS	11 12
***In vivo***		
Rat	SOD	13
Mouse	Free DNA, IL-6, IFN-γ, MMP-9, VEGF, TNF-α, p-AMPK, SR-A, MPO, NET-associated guanine histone H3	14
Mouse	Free DNA, IFN-γ, MPO-DNA complexes, TNF-α, IL-6, HIF-1α, MMP-9, p-AMPK, SR-A	15
Mouse	GPx4, ACSL4, CDKN1A	16
Rabbit	CD80/CD28, CD86/CD28, CD40/CD154 (CD40L), CD4, CD1d, myeloid differentiation primary response gene 88-like, IL-2, IL-10, IL-18, IFN-γ, COX-2	17

ACSL4: Acyl coenzyme A synthetase long chain family member 4; Bax: B-cell lymphoma 2-associated X protein; Bcl-2: B-cell lymphoma 2; cdc2: cell division cycle 2; CDK: cyclin-dependent kinase; CDKN: cyclin-dependent kinase inhibitor; COX-2: cyclooxygenase-2; GPx4: glutathione peroxidase 4; GSH: reduced glutathione; HIF-1α: hypoxia-inducible factor 1α; HMGB1: high-mobility group box-1; IFN: interferon; IL: interleukin; JAK2: Janus kinase 2; MDA: malondialdehyde; MPO: myeloperoxidase; NF-κB: nuclear factor kappa-light-chain-enhancer of activated B cells; O3: ozone; p-AMPK: phosphorylated adenosine monophosphate-activated protein kinase; PARP-1: poly(adenosine diphosphate ribose) polymerase-1; p-JNK: phosphorylated c-Jun N-terminal kinase; ROS: reactive oxygen species; SOD: superoxide dismutase; SR-A: class A scavenger receptor; STAT3: signal transducer and activator of transcription 3; TNF-α: tumor necrosis factor α; VEGF: vascular endothelial growth factor.

## Conclusions

A plethora of observational studies point to the beneficial action of O_3_ administration for several medical applications. However, the therapeutic efficacy of O_3_ is still a matter of debate within the medical community and regulatory authorities, likely due to the incomplete knowledge of the biological and molecular mechanisms involved.

In fact, the scientific literature on the experimental application of medical O_3_ is, until now, limited, and sometimes contradictory results have been published; this probably depends on the heterogeneity of the experimental models and gas concentrations used and of the administration procedures. Nevertheless, information is progressively growing on the cellular and molecular pathways that are stimulated by O_3_ and that could account for its observed positive effects.

In the current state of knowledge, the basic mechanism has been identified in the eustress induced by low-concentration O_3_, which activates a complex antioxidant response through Nrf2, promotes cell survival and inhibits apoptosis and autophagy. At low concentrations, O_3_ also exerts an anti-inflammatory effect by affecting cytokine expression, inhibiting the expression of MMPs and increasing macrophage phagocytic activity. In addition to the activation of antioxidant and anti-inflammatory pathways, the O_3_-driven stimulation of fibroblast, chondrocyte and osteoblast proliferation and the secretion of matrix components may explain the great regenerative potential demonstrated by O_3_. In contrast, inhibition of cell proliferation, invasion, and migration and pro-apoptotic effects have been reported in some cancer cell lines treated with O_3_; however, experimental data on the antitumor properties of O_3_ are lacking, and more extended and conclusive evidence is needed.

In this review, we did not consider some biological processes that might potentially be targeted by medical O_3_ administration, such as neuronal functionality, neuromuscular transmission and muscle contraction, or the enhancement of blood microcirculation (although some beneficial effects of O_3_ treatment were observed in clinical practice in orthopedics, neurology and regenerative medicine); in fact, experimental research on the effects of low-concentration O_3_ on these processes is still at a very early stage. Despite these limitations, this review provides a wide overview of the current knowledge on the biological mechanisms underlying the main therapeutic effects of low-concentration O_3_, thus representing an original compendium that is easy for scientists and clinicians to consult.

## Additional files:

***Additional Table 1:*** *Experimental investigations on the cellular and molecular mechanisms underlying the antioxidant effect of low-concentration O_3_.*

***Additional Table 2:*** *Molecular modulation of low-concentration O_3_ for antioxidant effects.*

***Additional Table 3:*** *Experimental investigations on the cellular and molecular mechanisms underlying the anti-inflammatory effect of low-concentration O_3_.*

***Additional Table 4:*** *Molecular modulation of low O_3_ concentrations for anti-inflammatory effects.*

***Additional Table 5:*** *Experimental investigations on the cellular and molecular mechanisms underlying the effects of low-concentration O_3_ on apoptosis and autophagy.*

***Additional Table 6:*** *Molecular modulation of low-concentration O_3_ on apoptosis and autophagy.*

***Additional Table 7:*** *Experimental investigations on the cellular and molecular mechanisms underlying the pro-regenerative effects of low-concentration O_3_.*

***Additional Table 8:*** *Molecular modulation of low-concentration O_3_ for regenerative effects.*

***Additional Table 9:*** *Experimental investigations on the cellular and molecular mechanisms underlying the anticancer effects of low-concentration O_3_.*

***Additional Table 10:*** *Molecular modulation of low-concentration O_3_ for anticancer effects.*

**Facts**

1. Low-concentration ozone (O_3_) induces eustress, activating a complex antioxidant response through nuclear factor erythroid 2-related factor 2.
2. Low-concentration O_3_ exerts anti-inflammatory effects by modulating cytokine expression, inhibiting metalloproteinases and activating macrophages.
3. Low-concentration O_3_ induces tissue regeneration by stimulating fibroblast, chondrocyte, and osteoblast activity.
4. Low-concentration O_3_ inhibits cancer cell proliferation and migration, and induces apoptosis.

**Open Questions**

1. What are the mechanisms underlying the effects of low-concentration O_3_ on cancer cells and tumors?
2. Does low-concentration O_3_ affect the membrane potential of nervous and muscle cells?
3. Does low-concentration O_3_ affect blood microcirculation?

## Data Availability

*All data generated or analyzed in this study are included in this published article and its additional files.*

## References

[1] Barbe A, Mikhailenko S, Starikova E, Tyuterev V (2022). High resolution infrared spectroscopy in support of ozone atmospheric monitoring and validation of the potential energy function. Molecules.

[2] Forbes RJ, Lefèbvre E, de Bruijn JG (1969). Martinus van marum: life and work.

[3] Schönbein CF (1840). On the odour accompanying electricity and on the probability of its dependence on the presence of a new substance. Philos Mag (Abingdon).

[4] Schönbein CF (1863). Ueber die katalytische Wirksamkeit organischer Materien und deren Verbreitung in der Pflanzen- und Thierwelt. J Prakt Chem.

[5] Schönbein CF (1851). On some secondary physiological effects produced by atmospheric electricity. Med Chir Trans.

[6] Fox CB (1873). Ozone and antozone: their history and nature. When, where, why, how is ozone observed in the atmosphere?.

[7] Kellogg JH (1879). Diphtheria: its causes, prevention, and proper treatment. Battle Creek.

[8] Bocci V, Zanardia I, Valacchi G, Borrelli E, Travagli V (2015). Validity of oxygen-ozone therapy as integrated medication form in chronic inflammatory diseases. Cardiovasc Hematol Disord Drug Targets.

[9] Oliveira Modena DA, de Castro Ferreira R, Froes PM, Rocha KC (2022). Ozone therapy for dermatological conditions: a systematic review. J Clin Aesthet Dermatol.

[10] Jeyaraman M, Jeyaraman N, Ramasubramanian S (2024). Ozone therapy in musculoskeletal medicine: a comprehensive review. Eur J Med Res.

[11] Hidalgo-Tallón FJ, Torres-Morera LM, Baeza-Noci J, Carrillo-Izquierdo MD, Pinto-Bonilla R (2022). Updated review on ozone therapy in pain medicine. Front Physiol.

[12] El Meligy OA, Elemam NM, Talaat IM (2023). Ozone therapy in medicine and dentistry: a review of the literature. Dent J (Basel).

[13] Veneri F, Filippini T, Consolo U, Vinceti M, Generali L (2024). Ozone therapy in dentistry: an overview of the biological mechanisms involved (Review). Biomed Rep.

[14] Sciorsci RL, Lillo E, Occhiogrosso L, Rizzo A (2020). Ozone therapy in veterinary medicine: a review. Res Vet Sci.

[15] Viebahn-Haensler R, León Fernández OS (2021). Ozone in Medicine. The low-dose ozone concept and its basic biochemical mechanisms of action in chronic inflammatory diseases. Int J Mol Sci.

[16] Viebahn-Hänsler R, León Fernández OS, Fahmy Z (2012). Ozone in medicine: the low-dose ozone concept—guidelines and treatment strategies. Ozone Sci Eng.

[17] Sagai M, Bocci V (2011). Mechanisms of action involved in ozone therapy: is healing induced via a mild oxidative stress?. Med Gas Res.

[18] Niki E (2016). Oxidative stress and antioxidants: distress or eustress?. Arch Biochem Biophys.

[19] Sies H (2017). Hydrogen peroxide as a central redox signaling molecule in physiological oxidative stress: oxidative eustress. Redox Biol.

[20] Crawford DR, Davies KJ (1994). Adaptive response and oxidative stress. Environ Health Perspect.

[21] Niki E (2009). Lipid peroxidation: physiological levels and dual biological effects. Free Radic Biol Med.

[22] Izadi M, Jafari-Oori M, Eftekhari Z (2024). Effect of ozone therapy on diabetes-related foot ulcer outcomes: a systematic review and meta-analysis. Curr Pharm Des.

[23] Liu J, Huang Y, Huang J, Yang W, Tao R (2025). Effects of ozone therapy as an adjuvant in the treatment of periodontitis: a systematic review and meta-analysis. BMC Oral Health.

[24] Rezaeianjam M, Khabazian A, Khabazian T (2025). Efficacy of ozone therapy in dentistry with approach of healing, pain management, and therapeutic outcomes: a systematic review of clinical trials. BMC Oral Health.

[25] Machado AU, Contri RV (2022). Effectiveness and safety of ozone therapy for dermatological disorders: a literature review of clinical trials. Indian J Dermatol.

[26] Liu L, Zeng L, Gao L, Zeng J, Lu J (2023). Ozone therapy for skin diseases: cellular and molecular mechanisms. Int Wound J.

[27] Masan J, Sramka M, Rabarova D (2021). The possibilities of using the effects of ozone therapy in neurology. Neuro Endocrinol Lett.

[28] Ronconi G, Mariantonietta A, Codazza S (2025). Effects of oxygen-ozone injections in upper limb disorders: scoping review. J Clin Med.

[29] Ghatge SB, Asarkar A, Warghade SS, Shirsat S, Deb A (2024). Ozone disc nucleolysis for cervical intervertebral disc herniation: a systematic review and meta-analysis. Cureus.

[30] Pecorelli A, Bocci V, Acquaviva A (2013). NRF2 activation is involved in ozonated human serum upregulation of HO-1 in endothelial cells. Toxicol Appl Pharmacol.

[31] Galiè M, Costanzo M, Nodari A (2018). Mild ozonisation activates antioxidant cell response by the Keap1/Nrf2 dependent pathway. Free Radic Biol Med.

[32] Siniscalco D, Trotta MC, Brigida AL (2018). Intraperitoneal administration of oxygen/ozone to rats reduces the pancreatic damage induced by streptozotocin. Biology (Basel).

[33] Ding S, Duanmu X, Xu L, Zhu L, Wu Z (2023). Ozone pretreatment alleviates ischemiareperfusion injury-induced myocardial ferroptosis by activating the Nrf2/Slc7a11/Gpx4 axis. Biomed Pharmacother.

[34] Zhu F, Ding S, Liu Y, Wang X, Wu Z (2024). Ozone-mediated cerebral protection: Unraveling the mechanism through ferroptosis and the NRF2/SLC7A11/GPX4 signaling pathway. J Chem Neuroanat.

[35] Tuo QZ, Zhang ST, Lei P (2022). Mechanisms of neuronal cell death in ischemic stroke and their therapeutic implications. Med Res Rev.

[36] Li J, Liu X, Wang Z (2024). Ozone therapy mitigates parthanatos after ischemic stroke. Biol Res.

[37] Guo HZ, Yu SL, Chen HW (2025). Major ozonated autoheamotherapy alleviates skeletal muscle ischemia/reperfusion injury by regulating Nrf2/HO-1 pathway. Kaohsiung J Med Sci.

[38] Qiu T, Wang ZS, Liu XH (2017). Effect of ozone oxidative preconditioning on oxidative stress injury in a rat model of kidney transplantation. Exp Ther Med.

[39] Yu G, Liu X, Chen Z (2016). Ozone therapy could attenuate tubulointerstitial injury in adenine-induced CKD rats by mediating Nrf2 and NF-κB. Iran J Basic Med Sci.

[40] Wang Z, Zhang A, Meng W (2018). Ozone protects the rat lung from ischemia-reperfusion injury by attenuating NLRP3-mediated inflammation, enhancing Nrf2 antioxidant activity and inhibiting apoptosis. Eur J Pharmacol.

[41] Mallok A, Vaillant JD, Soto MT (2015). Ozone protective effects against PTZ-induced generalized seizures are mediated by reestablishment of cellular redox balance and A1 adenosine receptors. Neurol Res.

[42] León Fernández OS, Ajamieh HH, Berlanga J (2008). Ozone oxidative preconditioning is mediated by A1 adenosine receptors in a rat model of liver ischemia/ reperfusion. Transpl Int.

[43] Chen H, Xing B, Liu X (2008). Ozone oxidative preconditioning protects the rat kidney from reperfusion injury: the role of nitric oxide. J Surg Res.

[44] Chen H, Xing B, Liu X (2008). Similarities between ozone oxidative preconditioning and ischemic preconditioning in renal ischemia/reperfusion injury. Arch Med Res.

[45] Ozkan H, Ekinci S, Uysal B (2015). Evaluation and comparison of the effect of hypothermia and ozone on ischemia-reperfusion injury of skeletal muscle in rats. J Surg Res.

[46] Wang L, Chen Z, Liu Y, Du Y, Liu X (2018). Ozone oxidative postconditioning inhibits oxidative stress and apoptosis in renal ischemia and reperfusion injury through inhibition of MAPK signaling pathway. Drug Des Devel Ther.

[47] Madej P, Plewka A, Madej JA (2007). Ozone therapy in induced endotoxemic shock. II. The effect of ozone therapy upon selected histochemical reactions in organs of rats in endotoxemic shock. Inflammation.

[48] Nasezadeh P, Shahi F, Fridoni M, Seydi E, Izadi M, Salimi A (2017). Moderate O(3)/O(2) therapy enhances enzymatic and non-enzymatic antioxidant in brain and cochlear that protects noise-induced hearing loss. Free Radic Res.

[49] Inguscio CR, Dalla Pozza E, Dando I (2023). Mitochondrial features of mouse myoblasts are finely tuned by low doses of ozone: the evidence in vitro. Int J Mol Sci.

[50] Oliveira MM, Correia S, Peirone C, Magalhães M, Oliveira P, Peixoto F (2024). Impact of ozone therapy on mouse liver mitochondrial function and antioxidant system. Biochimie.

[51] Bocci V, Valacchi G, Corradeschi F (1998). Studies on the biological effects of ozone: 7. Generation of reactive oxygen species (ROS) after exposure of human blood to ozone. J Biol Regul Homeost Agents.

[52] Bocci V, Aldinucci C (2006). Biochemical modifications induced in human blood by oxygenation-ozonation. J Biochem Mol Toxicol.

[53] Travagli V, Zanardi I, Bernini P, Nepi S, Tenori L, Bocci V (2010). Effects of ozone blood treatment on the metabolite profile of human blood. Int J Toxicol.

[54] Ciborowski M, Lipska A, Godzien J (2012). Combination of LC-MS- and GC-MS-based metabolomics to study the effect of ozonated autohemotherapy on human blood. J Proteome Res.

[55] Inguscio CR, Cisterna B, Carton F, Barberis E, Manfredi M, Malatesta M (2023). Modifications of blood molecular components after treatment with low ozone concentrations. Int J Mol Sci.

[56] Cho KH, Kim JE, Lee MS, Bahuguna A (2024). Oral supplementation of ozonated sunflower oil augments plasma antioxidant and anti-inflammatory abilities with enhancement of high-density lipoproteins functionality in rats. Antioxidants (Basel).

[57] Candelario-Jalil E, Mohammed-Al-Dalain S, Fernández OS (2001). Oxidative preconditioning affords protection against carbon tetrachloride-induced glycogen depletion and oxidative stress in rats. J Appl Toxicol.

[58] Guanche D, Hernandez F, Zamora Z, Alonso Y (2010). Effect of ozone pre-conditioning on redox activity in a rat model of septic shock. Toxicol Mech Methods.

[59] Gultekin FA, Bakkal BH, Guven B (2013). Effects of ozone oxidative preconditioning on radiation-induced organ damage in rats. J Radiat Res.

[60] Aksu F, Kavaklı A, Kuloglu T (2024). Use of ozone therapy and thymoquinone in the prevention of formaldehyde toxicity by inhalation: an experimental study. Cureus.

[61] León OS, Menéndez S, Merino N (1998). Ozone oxidative preconditioning: a protection against cellular damage by free radicals. Mediators Inflamm.

[62] Kaya E, Yilmaz S, Aksu F, Kavaklı A (2024). Evaluation of the effects of overdose Acetaminophen toxicity in rats: ozone, which can be preferred as a complementary therapy. Rev Cient.

[63] Borrego A, Zamora ZB, González R (2004). Protection by ozone preconditioning is mediated by the antioxidant system in cisplatin-induced nephrotoxicity in rats. Mediators Inflamm.

[64] Calunga JL, Trujillo Y, Menéndez S (2009). Ozone oxidative post-conditioning in acute renal failure. J Pharm Pharmacol.

[65] Caliskan B, Guven A, Ozler M (2011). Ozone therapy prevents renal inflammation and fibrosis in a rat model of acute pyelonephritis. Scand J Clin Lab Invest.

[66] Uğuz S, Demirer Z, Uysal B (2016). Medical ozone therapy reduces shock wave therapy-induced renal injury. Ren Fail.

[67] Delgado-Roche L, Hernández-Matos Y, Medina EA, Morejón D, González MR, Martínez-Sánchez G (2014). Ozone-oxidative preconditioning prevents doxorubicin-induced cardiotoxicity in sprague-dawley rats. Sultan Qaboos Univ Med J.

[68] Tasdemir S, Tasdemir C, Vardi N (2013). Effects of ozone therapy on cyclophosphamide-induced urinary bladder toxicity in rats. Clin Invest Med.

[69] Salem EA, Salem NA, Hellstrom WJ (2017). Therapeutic effect of ozone and rutin on adriamycin-induced testicular toxicity in an experimental rat model. Andrologia.

[70] Aydogdu I, Ilbey YO, Coban G (2019). Does ozone administration have a protective or therapeutic effect against radiotherapy-induced testicular injury?. J Cancer Res Ther.

[71] Khorsandi L, Varaa N, Dadfar R (2024). The protective effect of ozone on the mice testicular damage induced by methotrexate. JBRA Assist Reprod.

[72] Kesik V, Uysal B, Kurt B, Kismet E, Koseoglu V (2009). Ozone ameliorates methotrexate-induced intestinal injury in rats. Cancer Biol Ther.

[73] Abu-Gharbieh E, Bayoumi FA, Ahmed NG (2014). Alleviation of antioxidant defense system by ozonized olive oil in DNBS-induced colitis in rats. Mediators Inflamm.

[74] Zamora Rodríguez ZB, González Alvarez R, Guanche D (2007). Antioxidant mechanism is involved in the gastroprotective effects of ozonized sunflower oil in ethanol-induced ulcers in rats. Mediators Inflamm.

[75] Guven A, Gundogdu G, Sadir S (2008). The efficacy of ozone therapy in experimental caustic esophageal burn. J Pediatr Surg.

[76] Al-Dalain SM, Martínez G, Candelario-Jalil E (2001). Ozone treatment reduces markers of oxidative and endothelial damage in an experimental diabetes model in rats. Pharmacol Res.

[77] Uysal B, Yasar M, Ersoz N (2010). Efficacy of hyperbaric oxygen therapy and medical ozone therapy in experimental acute necrotizing pancreatitis. Pancreas.

[78] Kaldirim U, Uysal B, Yuksel R (2014). Ozone therapy ameliorates paraquat-induced lung injury in rats. Exp Biol Med (Maywood).

[79] Erken HA, Genç O, Erken G, Ayada C, Gündoğdu G, Doğan H (2015). Ozone partially prevents diabetic neuropathy in rats. Exp Clin Endocrinol Diabetes.

[80] Biçer Ş, Sayar İ, Gürsul C (2016). Use of ozone to treat ileostomy dermatitis in an experimental rat model. Med Sci Monit.

[81] Magenta A, D’Agostino M, Sileno S (2019). The oxidative stress-induced miR-200c is upregulated in psoriasis and correlates with disease severity and determinants of cardiovascular risk. Oxid Med Cell Longev.

[82] Zhang L, Men X, Yu S (2022). Ozone protects cardiomyocytes from myocardial ischemia-reperfusion injury through miR-200c/FOXO3 axis. J Recept Signal Transduct Res.

[83] Ogut E, Yildirim FB, Sarikcioglu L, Aydin MA, Demir N (2020). Neuroprotective effects of ozone therapy after sciatic nerve cut injury. Kurume Med J.

[84] Xia L, Sun Y, Zhou Y, Yang Q, Huang J, Liu D (2024). Major ozonated autohemotherapy promoted functional recovery following spinal cord injury in adult rats via the inhibition of oxidative stress and inflammation. Open Life Sci.

[85] Cho KH, Kim JE, Bahuguna A, Kang DJ (2023). Ozonated sunflower oil exerted potent anti-inflammatory activities with enhanced wound healing and tissue regeneration abilities against acute toxicity of carboxymethyllysine in zebrafish with improved blood lipid profile. Antioxidants (Basel).

[86] Cabral IL, Utzig SL, Banhuk FW (2020). Aqueous ozone therapy improves the standard treatment of leishmaniasis lesions in animals leading to local and systemic alterations. Parasitol Res.

[87] Cabral IL, Lima LBS, Três DP (2025). Ozonated sunflower oil stimulates the local antioxidant system and helps meglumine antimoniate to ameliorate cutaneous leishmaniasis lesions. ACS Omega.

[88] Cisterna B, Costanzo M, Nodari A (2020). Ozone activates the Nrf2 pathway and improves preservation of explanted adipose tissue in vitro. Antioxidants (Basel).

[89] Safwat MH, El-Sawalhi MM, Mausouf MN, Shaheen AA (2014). Ozone ameliorates age-related oxidative stress changes in rat liver and kidney: effects of pre- and post-ageing administration. Biochemistry (Mosc).

[90] Shehata NI, Abd-Elgawad HM, Mawsouf MN, Shaheen AA (2012). The potential role of ozone in ameliorating the age-related biochemical changes in male rat cerebral cortex. Biogerontology.

[91] El-Sawalhi MM, Darwish HA, Mausouf MN, Shaheen AA (2013). Modulation of age-related changes in oxidative stress markers and energy status in the rat heart and hippocampus: a significant role for ozone therapy. Cell Biochem Funct.

[92] Elkholy WB, Al-Gholam MA (2018). Role of medical ozone in attenuating age-related changes in the rat cerebellum. Microscopy (Oxf).

[93] Chen Z, Liu X, Yu G (2016). Ozone therapy ameliorates tubulointerstitial inflammation by regulating TLR4 in adenine-induced CKD rats. Ren Fail.

[94] Zhu L, Ding S, Xu L, Wu Z (2022). Ozone treatment alleviates brain injury in cerebral ischemic rats by inhibiting the NF-κB signaling pathway and autophagy. Cell Cycle.

[95] Xu L, Wang C, Zou Z, Wu Z (2021). Ozone attenuated H9c2 cell injury induced by doxorubicin. J Cardiovasc Pharmacol.

[96] Simonetti V, Franzini M, Iaffaioli RV, Pandolfi S, Valdenassi L, Quagliariello V (2018). Anti-inflammatory effects of ozone in human melanoma cells and its modulation of tumour microenvironment. Int J of Adv Res.

[97] Wang Z, Han Q, Guo YL, Liu XH, Qiu T (2018). Effect of ozone oxidative preconditioning on inflammation and oxidative stress injury in rat model of renal transplantation. Acta Cir Bras.

[98] Sun W, Pei L (2012). Ozone preconditioning and exposure to ketamine attenuates hepatic inflammation in septic rats. Arch Med Sci.

[99] Kucukgul A, Erdogan S, Gonenci R, Ozan G (2016). Beneficial effects of nontoxic ozone on H(2)O(2)-induced stress and inflammation. Biochem Cell Biol.

[100] Yan C, Zhang Y, Jin L (2024). Medical ozone alleviates acute lung injury by enhancing phagocytosis targeting NETs via AMPK/SR-A1 axis. J Biomed Res.

[101] Kim SY, Lee JO, Lee S (2024). Ozonated sunflower oil (OSO) alleviates inflammatory responses in oxazolone-induced atopic dermatitis (AD)-like mice and LPS-treated RAW 264.7 cells. J Microbiol Biotechnol.

[102] Yu S, Guo H, Luo Y, Chen H (2021). Ozone protects cardiomyocytes against ischemia/reperfusion injury: Regulating the heat shock protein 70 (HPS70) expression through activating the JAK2/STAT3 pathway. Bioengineered.

[103] Yu G, Bai Z, Chen Z (2017). The NLRP3 inflammasome is a potential target of ozone therapy aiming to ease chronic renal inflammation in chronic kidney disease. Int Immunopharmacol.

[104] Xu G, Sun X, An J, Sun F, Zhang C, Williams JP (2025). Ozone protects from myocardial ischemia-reperfusion injury via inhibition of the NLRP3 inflammasome. Eur J Pharmacol.

[105] Wang P, Zhao Q, Zhu X, Cao S, Williams JP, An J (2025). Ozone therapy ameliorates LPS-induced acute lung injury in mice by inhibiting the NLRP3/ASC/CASPASE-1 axis. Shock.

[106] Wang D, Liu Y, Zong X, Yan S, Lu J (2023). Ozonated triglyceride protects against septic lethality via preventing the activation of NLRP3 inflammasome. Zhong Nan Da Xue Xue Bao Yi Xue Ban.

[107] Güçlü A, Erken HA, Erken G (2016). The effects of ozone therapy on caspase pathways, TNF-α, and HIF-1α in diabetic nephropathy. Int Urol Nephrol.

[108] Bertuccio MP, Rizzo V, Arena S (2023). Ozoile reduces the LPS-induced inflammatory response in colonic epithelial cells and THP-1 monocytes. Curr Issues Mol Biol.

[109] Armeli F, Mengoni B, Menin M (2025). Ozone saline solution polarizes microglial cells towards an anti-inflammatory phenotype. Molecules.

[110] Cisterna B, Costanzo M, Lacavalla MA (2021). Low ozone concentrations differentially affect the structural and functional features of non-activated and activated fibroblasts in vitro. Int J Mol Sci.

[111] Lacavalla MA, Inguscio CR, Cisterna B (2022). Ozone at low concentration modulates microglial activity in vitro: A multimodal microscopy and biomolecular study. Microsc Res Tech.

[112] Bocci V, Paulesu L (1990). Studies on the biological effects of ozone 1. Induction of interferon gamma on human leucocytes. Haematologica.

[113] Paulesu L, Luzzi E, Bocci V (1991). Studies on the biological effects of ozone: 2. Induction of tumor necrosis factor (TNF-alpha) on human leucocytes. Lymphokine Cytokine Res.

[114] Larini A, Bocci V (2005). Effects of ozone on isolated peripheral blood mononuclear cells. Toxicol In Vitro.

[115] Valacchi G, Bocci V (1999). Studies on the biological effects of ozone: 10. Release of factors from ozonated human platelets. Mediators Inflamm.

[116] Inguscio CR, Cisterna B, Lacavalla MA (2023). Ozone and procaine increase secretion of platelet-derived factors in platelet-rich plasma. Eur J Histochem.

[117] Bocci V, Luzzi E, Corradeschi F, Paulesu L (1993). Studies on the biological effects of ozone: 5. Evaluation of immunological parameters and tolerability in normal volunteers receiving ambulatory autohaemotherapy. Biotherapy.

[118] Valacchi G, Bocci V (2000). Studies on the biological effects of ozone: 11. Release of factors from human endothelial cells. Mediators Inflamm.

[119] Simonetti V, Quagliariello V, Giustetto P, Franzini M, Iaffaioli RV (2017). Association of ozone with 5-fluorouracil and cisplatin in regulation of human colon cancer cell viability: in vitro anti-inflammatory properties of ozone in colon cancer cells exposed to lipopolysaccharides. Evid Based Complement Alternat Med.

[120] Cho KH, Kim JE, Bahuguna A, Kang DJ (2023). Long-term supplementation of ozonated sunflower oil improves dyslipidemia and hepatic inflammation in hyperlipidemic zebrafish: suppression of oxidative stress and inflammation against carboxymethyllysine toxicity. Antioxidants (Basel).

[121] Kato Y, Sakoh M, Nagai T (2024). Ozonated olive oil intake attenuates hepatic steatosis in obese db/db mice. J Oleo Sci.

[122] Chen H, Xing B, Liu X (2008). Ozone oxidative preconditioning inhibits inflammation and apoptosis in a rat model of renal ischemia/reperfusion injury. Eur J Pharmacol.

[123] Sancak EB, Turkön H, Çukur S (2016). Major ozonated autohemotherapy preconditioning ameliorates kidney ischemia-reperfusion injury. Inflammation.

[124] Vaillant JD, Fraga A, Díaz MT (2013). Ozone oxidative postconditioning ameliorates joint damage and decreases pro-inflammatory cytokine levels and oxidative stress in PG/PS-induced arthritis in rats. Eur J Pharmacol.

[125] Tartari APS, Moreira FF, Pereira M (2020). Anti-inflammatory effect of ozone therapy in an experimental model of rheumatoid arthritis. Inflammation.

[126] Yamanel L, Kaldirim U, Oztas Y (2011). Ozone therapy and hyperbaric oxygen treatment in lung injury in septic rats. Int J Med Sci.

[127] Aslaner A, Çakır T, Tekeli S (2016). Medical ozone treatment ameliorates the acute distal colitis in rat. Acta Cir Bras.

[128] Uysal B, Demirbag S, Poyrazoglu Y (2012). Medical ozone therapy decreases postoperative uterine adhesion formation in rats. Arch Gynecol Obstet.

[129] Wei A, Feng H, Jia XM, Tang H, Liao YY, Li BR (2018). Ozone therapy ameliorates inflammation and endometrial injury in rats with pelvic inflammatory disease. Biomed Pharmacother.

[130] Suzuki N, Hirano M, Shinozuka Y, Kawai K, Okamoto Y, Isobe N (2022). Effects of ozonized glycerin on inflammation of mammary glands induced by intramammary lipopolysaccharide infusion in goats. Anim Sci J.

[131] Xie TY, Yan W, Lou J, Chen XY (2016). Effect of ozone on vascular endothelial growth factor (VEGF) and related inflammatory cytokines in rats with diabetic retinopathy. Genet Mol Res.

[132] Kaya A, Sonmez M, Kar T (2017). Efficiency of ozone therapy in a rat model of experimental uveitis. Ocul Immunol Inflamm.

[133] Lu J, Chen M, Gao L (2018). A preliminary study on topical ozonated oil in the therapeutic management of atopic dermatitis in murine. J Dermatolog Treat.

[134] Salem NA, Assaf N, Ismail MF, Khadrawy YA, Samy M (2016). Ozone therapy in ethidium bromide-induced demyelination in rats: possible protective effect. Cell Mol Neurobiol.

[135] Gultekin FA, Cakmak GK, Turkcu UO, Yurdakan G, Demir FE, Comert M (2013). Effects of ozone oxidative preconditioning on liver regeneration after partial hepatectomy in rats. J Invest Surg.

[136] Wang Q, Liu CZ, Li BT (2025). Ozone controls the metabolism of tryptophan protecting against sepsis-induced intestinal damage by activating aryl hydrocarbon receptor. World J Gastroenterol.

[137] Erel S, Küçükk A, Dikmen K (2024). Evaluation of the efficacy of ozone therapy on liver tissue in the treatment of sepsis in rats with cecal perforation. Medicina (Kaunas).

[138] Hao K, Li Y, Feng J (2015). Ozone promotes regeneration by regulating the inflammatory response in zebrafish. Int Immunopharmacol.

[139] Sahin H, Simsek T, Turkon H (2016). The acute effects of preoperative ozone theraphy on surgical wound healing. Acta Cir Bras.

[140] Çakır T, Aslaner A, Tekeli S (2016). Effect of ozone on colon anastomoses in rat peritonitis model. Acta Cir Bras.

[141] Prajoko YW, Manapa CH, Nugroho TN (2025). Ozonated aloe vera oil accelerates radiation dermatitis healing in sprague dawley rats. Middle East J Cancer.

[142] Kesik V, Yuksel R, Yigit N (2016). Ozone ameliorates doxorubicine-induced skin necrosis - results from an animal model. Int J Low Extrem Wounds.

[143] Gürkan G, Sayin M, Kizmazoglu C (2020). Evaluation of the neuroprotective effects of ozone in an experimental spine injury model. J Neurosurg Spine.

[144] Pivotto AP, de Souza Lima LB, Michelon A (2024). Topical application of ozonated sunflower oil accelerates the healing of lesions of cutaneous leishmaniasis in mice under meglumine antimoniate treatment. Med Microbiol Immunol.

[145] Wu H, Wang J, Lin Y (2024). Injectable ozone-rich nanocomposite hydrogel loaded with d-mannose for anti-inflammatory and cartilage protection in osteoarthritis treatment. Small.

[146] Murphy K, Elias G, Steppan J (2016). Percutaneous treatment of herniated lumbar discs with ozone: investigation of the mechanisms of action. J Vasc Interv Radiol.

[147] Wang J, Wu M, Lin X, Li Y, Fu Z (2018). Low-concentration oxygen/ozone treatment attenuated radiculitis and mechanical allodynia via PDE2A-cAMP/cGMP-NF-κB/p65 signaling in chronic radiculitis rats. Pain Res Manag.

[148] Lu L, Pan C, Chen L (2017). AMPK activation by peri-sciatic nerve administration of ozone attenuates CCI-induced neuropathic pain in rats. J Mol Cell Biol.

[149] Fan W, Liu C, Chen D (2023). Ozone alleviates MSU-induced acute gout pain via upregulating AMPK/GAS6/MerTK/SOCS3 signaling pathway. J Transl Med.

[150] Zhang XT, Zong LJ, Jia RM (2023). Ozone attenuates chemotherapy-induced peripheral neuropathy via upregulating the AMPK-SOCS3 axis. J Cancer Res Ther.

[151] Zhang W, Wang F, Zhang L, Sun T, Fu Z (2021). Intrathecal injection of ozone alleviates CCI-induced neuropathic pain via the GluR6-NF-κB/p65 signalling pathway in rats. Mol Med Rep.

[152] Fuccio C, Luongo C, Capodanno P (2009). A single subcutaneous injection of ozone prevents allodynia and decreases the over-expression of pro-inflammatory caspases in the orbito-frontal cortex of neuropathic mice. Eur J Pharmacol.

[153] Jiang B, Su Y, Chen Q (2020). Protective effects of ozone oxidative postconditioning on long-term injury after renal ischemia/reperfusion in rat. Transplant Proc.

[154] Ruan S, Jia R, Hu L (2024). Ozone promotes macrophage efferocytosis and alleviates neuropathic pain by activating the AMPK/Gas6-MerTK/SOCS3 signaling pathway. Front Immunol.

[155] Huang N, Gao D, Zhou S, Huang Z (2023). Ozone reduces ischemic damage after a stroke by regulating the autophagy of astrocytes. Ann Transl Med.

[156] Xu L, Zhu L, Liu P, Wu Z, Zou Z (2021). Ozone induces tolerance against cardiomyocytes oxygen-glucose deprivation/reperfusion through inhibition of autophagy pathway. Exp Ther Med.

[157] Wu MY, Xing CY, Wang JN, Li Y, Lin XW, Fu ZJ (2018). Therapeutic dosage of ozone inhibits autophagy and apoptosis of nerve roots in a chemically induced radiculoneuritis rat model. Eur Rev Med Pharmacol Sci.

[158] Xu W, Zhao X, Sun P, Zhang C, Fu Z, Zhou D (2020). The effect of medical ozone treatment on cartilage chondrocyte autophagy in a rat model of osteoarthritis. Am J Transl Res.

[159] Sun P, Xu W, Zhao X (2022). Ozone induces autophagy by activating PPARγ/mTOR in rat chondrocytes treated with IL-1β. J Orthop Surg Res.

[160] Valacchi G, Lim Y, Belmonte G (2011). Ozonated sesame oil enhances cutaneous wound healing in SKH1 mice. Wound Repair Regen.

[161] Pchepiorka R, Moreira MS, Lascane N (2020). Effect of ozone therapy on wound healing in the buccal mucosa of rats. Arch Oral Biol.

[162] Karakaya E, Akdur A, Ayvazoğlu Soy E (2021). Effect of subcutaneous topical ozone therapy on second-degree burn wounds in rats: an experimental study. J Burn Care Res.

[163] Kovach I, Kravchenko L, Khotimska Y, Nazaryan R, Gargin V (2017). Influence of ozone therapy on oral tissue in modeling of chronic recurrent aphthous stomatitis. Georgian Med News.

[164] Martusevich AK, Struchkov AA, Nazarov VV, Fedotova AS, Artamonov MY, Peretyagin SP (2022). Estimation of effectiveness of local reactive oxygen species for stimulation of regenerative processes in experimental burn wound. Appl Sci.

[165] Aktas S, Ozsunar Y, Ogut S, Gokce S, Tataroglu C (2023). Comparative efficacy of red beetroot extract and ozonated olive oil on wound healing in rats. J Coll Physicians Surg Pak.

[166] Alpan AL, Toker H, Ozer H (2016). Ozone Therapy enhances osseous healing in rats with diabetes with calvarial defects: a morphometric and immunohistochemical study. J Periodontol.

[167] Miyasawa EM, Ervolino E, Cardoso JM (2024). Effects of systemic ozone administration on the fresh extraction sockets healing: a histomorphometric and immunohistochemical study in rats. J Appl Oral Sci.

[168] Silva W, Santos J, Souza MC (2024). Ozone therapy improves early stages of osseointegration in ovariectomized rats. J Appl Oral Sci.

[169] Ozbay I, Ital I, Kucur C (2017). Effects of ozone therapy on facial nerve regeneration. Braz J Otorhinolaryngol.

[170] Kızılay Z, Kahraman Çetin N, Aksel M (2019). Ozone partially decreases axonal and myelin damage in an experimental sciatic nerve injury model. J Invest Surg.

[171] Ayık B, Ortadeveci A, Bakılan F (2024). Comparison of the effects of perineural and intraperitoneal ozone therapy on nerve healing in an experimental sciatic nerve injury model. Medicina (Kaunas).

[172] Ozturk O, Tezcan AH, Adali Y (2016). Effect of ozone and methylprednisolone treatment following crush type sciatic nerve injury. Acta Cir Bras.

[173] Lima Neto TJ, Delanora LA, ME SS (2024). Ozone improved bone dynamic of female rats using zoledronate. Tissue Eng Part C Methods.

[174] Kan B, Sencimen M, Bayar GR (2015). Histomorphometric and microtomographic evaluation of the effects of hyperbaric oxygen and systemic ozone, used alone and in combination, on calvarial defect healing in rats. J Oral Maxillofac Surg.

[175] Uslu K, Tansuker HD, Tabaru A (2020). Investigation of the effects of thrombocyte-rich plasma, systemic ozone and hyperbaric oxygen treatment on intraoral wound healing in rats: experimental study. Eur Arch Otorhinolaryngol.

[176] Gurger M, Once G, Yilmaz E (2021). The effect of the platelet-rich plasma and ozone therapy on tendon-to-bone healing in the rabbit rotator cuff repair model. J Orthop Surg Res.

[177] Borges G, Elias ST, da Silva SM (2017). In vitro evaluation of wound healing and antimicrobial potential of ozone therapy. J Craniomaxillofac Surg.

[178] Xiao W, Tang H, Wu M (2017). Ozone oil promotes wound healing by increasing the migration of fibroblasts via PI3K/Akt/mTOR signaling pathway. Biosci Rep.

[179] Soares CD, Morais TML, Araújo R (2019). Effects of subcutaneous injection of ozone during wound healing in rats. Growth Factors.

[180] Valacchi G, Zanardi I, Lim Y (2013). Ozonated oils as functional dermatological matrices: effects on the wound healing process using SKH1 mice. Int J Pharm.

[181] Valacchi G, Sticozzi C, Zanardi I (2016). Ozone mediators effect on “in vitro” scratch wound closure. Free Radic Res.

[182] Kim HS, Noh SU, Han YW (2009). Therapeutic effects of topical application of ozone on acute cutaneous wound healing. J Korean Med Sci.

[183] Taşdöven İ, Emre AU, Gültekin FA (2019). Effects of ozone preconditioning on recovery of rat colon anastomosis after preoperative radiotherapy. Adv Clin Exp Med.

[184] Haj B, Sukhotnik I, Shaoul R (2014). Effect of ozone on intestinal recovery following intestinal ischemia-reperfusion injury in a rat. Pediatr Surg Int.

[185] Huang P, Wang R, Pang X, Yang Y, Guan Y, Zhang D (2022). Platelet-rich plasma combined with ozone prevents cartilage destruction and improves weight-bearing asymmetry in a surgery-induced osteoarthritis rabbit model. Ann Palliat Med.

[186] Unlu E, Gül Satar NY, Onguncan O, Güler S, Uslu N, Ukum MO (2025). Chondroprotective effects of ozone and hyaluronic acid in rat knee osteoarthritis: Comparison of intra-articular and systemic administration. Res Vet Sci.

[187] Asadi S, Farzanegi P, Azarbayjani MA (2020). Combined therapies with exercise, ozone and mesenchymal stem cells improve the expression of HIF1 and SOX9 in the cartilage tissue of rats with knee osteoarthritis. Physiol Int.

[188] Baggio AMP, Bizelli VF, Delamura IF (2024). Systemic ozone therapy as an adjunctive treatment in guided bone regeneration: a histomorphometrical and immunohistochemical study in rats. Clin Oral Investig.

[189] Yılmaz O, Erken HY, İçkin Gülen M, Güven Bağla A, Adalı Y, Kuru T (2025). The effects of systemic ozone application and hyperbaric oxygen therapy on avascular necrosis of the femoral head: an experimental study in the vascular deprivation of the rat femoral head model. Jt Dis Relat Surg.

[190] Krkl C, Yiğit MV, Özercan İ H, Aygen E, Gültürk B, Artaş G (2016). The effect of ozonated olive oil on neovascularizatıon in an experimental skin flap model. Adv Skin Wound Care.

[191] Atashi F, Modarressi A, Pepper MS (2015). The role of reactive oxygen species in mesenchymal stem cell adipogenic and osteogenic differentiation: a review. Stem Cells Dev.

[192] Castro JP, Grune T, Speckmann B (2016). The two faces of reactive oxygen species (ROS) in adipocyte function and dysfunction. Biol Chem.

[193] Costanzo M, Boschi F, Carton F (2018). Low ozone concentrations promote adipogenesis in human adipose-derived adult stem cells. Eur J Histochem.

[194] Kang KA, Hyun JW (2017). Oxidative stress, Nrf2, and epigenetic modification contribute to anticancer drug resistance. Toxicol Res.

[195] Hammad M, Raftari M, Cesário R (2023). Roles of oxidative stress and Nrf2 signaling in pathogenic and non-pathogenic cells: a possible general mechanism of resistance to therapy. Antioxidants (Basel).

[196] Karagülle OO, Yurttaş AG (2023). Synergistic effects of ozone with doxorubicin on the proliferation, apoptosis and metastatic profile of luminal-B type human breast cancer cell line. Tissue Cell.

[197] Costanzo M, Romeo A, Cisterna B (2020). Ozone at low concentrations does not affect motility and proliferation of cancer cells in vitro. Eur J Histochem.

[198] Inguscio CR, Carton F, Cisterna B (2024). Low ozone concentrations do not exert cytoprotective effects on tamoxifen-treated breast cancer cells in vitro. Eur J Histochem.

[199] Li J, Zeng T, Tang S (2021). Medical ozone induces proliferation and migration inhibition through ROS accumulation and PI3K/AKT/NF-κB suppression in human liver cancer cells in vitro. Clin Transl Oncol.

[200] Tang S, Xu B, Li J (2021). Ozone induces BEL7402 cell apoptosis by increasing reactive oxygen species production and activating JNK. Ann Transl Med.

[201] Tang S, Xu B, Pang H, Xiao L, Mei Q, He X (2023). Ozonated water inhibits hepatocellular carcinoma invasion and metastasis by regulating the HMGB1/NF-κB/STAT3 signaling pathway. J Hepatocell Carcinoma.

[202] Yıldırım M, Erkişi S, Yılmaz H (2022). The apoptotic effect of ozone therapy on mitochondrial activity of highly metastatic breast cancer cell line MDA-MB-231 using in vitro approaches. J Interv Med.

[203] Cannizzaro A, Verga Falzacappa CV, Martinelli M, Misiti S, Brunetti E, Bucci B (2007). O(2/3) exposure inhibits cell progression affecting cyclin B1/cdk1 activity in SK-N-SH while induces apoptosis in SK-N-DZ neuroblastoma cells. J Cell Physiol.

[204] Luongo M, Marinelli O, Zeppa L (2020). Cannabidiol and oxygen-ozone combination induce cytotoxicity in human pancreatic ductal adenocarcinoma cell lines. Cancers (Basel).

[205] Izzotti A, Fracchia E, Rosano C (2022). Efficacy of high-ozonide oil in prevention of cancer relapses mechanisms and clinical evidence. Cancers (Basel).

[206] Kendirci-Katirci R, Katirci E, Onal T, Vatansever HS (2025). The effects of ozone exposure on apoptotic mechanisms in Colo-320 and Colo-741 cell lines: implications for cancer therapy. J Mol Histol.

[207] Karagulle OO, Yurttas AG (2022). Ozone combined with doxorubicin exerts cytotoxic and anticancer effects on Luminal-A subtype human breast cancer cell line. Rev Assoc Med Bras (1992).

[208] An SJ, Kashiwakura JI, Katsuyama A (2025). Ozonated olive oil inhibits melanoma proliferation by inducing ferroptosis. Biochem Biophys Rep.

[209] Yalçın Y, Tekin İ, Tığlı Aydın RS (2022). Ionizing radiation induced DNA damage via ROS production in nano ozonized oil treated B-16 melanoma and OV-90 ovarian cells. Biochem Biophys Res Commun.

[210] Han F, Guo J, Mu M (2023). Mechanism of ozone alleviation of malignant ascites in hepatocellular carcinoma through the inhibition of neutrophil extracellular traps. PNAS Nexus.

[211] Rossmann A, Mandic R, Heinis J (2014). Intraperitoneal oxidative stress in rabbits with papillomavirus-associated head and neck cancer induces tumoricidal immune response that is adoptively transferable. Clin Cancer Res.

[212] Teke K, Ozkan TA, Cebeci OO (2017). Preventive effect of intravesical ozone supplementation on n-methyl-n-nitrosourea-induced non-muscle invasive bladder cancer in male rats. Exp Anim.

[213] Guo J, Guo J, Cheng B (2024). Ozone enhances the efficacy of radiation therapy in esophageal cancer. J Radiat Res.

[214] Kızıltan H, Bayir AG, Yucesan G (2015). Medical ozone and radiotherapy in a peritoneal, Erlich-ascites, tumor-cell model. Altern Ther Health Med.

[215] Dogan R, Hafız AM, Kiziltan HS (2018). Effectiveness of radiotherapy+ozone on tumoral tissue and survival in tongue cancer rat model. Auris Nasus Larynx.

[216] Zhang Y, Zhang C, Wu B, Li C, Lin J, Huang P (2023). Thermoresponsive ozone-enriched spray gel for postsurgical treatment of hepatocellular carcinoma. ACS Nano.

[217] Lu J, Zheng R, Shi Z (2024). Intracellular Criegee’s mechanism-based synergistic ozone therapy mediated by oleogels for cancer treatment. J Control Release.

[218] Chen M, Song L, Zhou Y (2025). Promotion of triple negative breast cancer immunotherapy by combining bioactive radicals with immune checkpoint blockade. Acta Biomater.

[219] Xu T, Zheng D, Chen M (2025). A cisplatin prodrug-based self-assembling ozone delivery nanosystem sensitizes radiotherapy in triple-negative breast cancer. Acta Pharm Sin B.

